# Dental Follicle-Derived Mesenchymal Stem Cell Exosome-Loaded Three-Dimensional Electrospun Poly(ε-caprolactone)/Gelatin Scaffold Accelerates Diabetic Foot Wound Healing

**DOI:** 10.1021/acsomega.5c11741

**Published:** 2026-04-20

**Authors:** Hulya Kara Subasat, Deniz Genc, Osman Bulut, Leyla Tekin, Ozay Eroglu, Hanife Sevval Dere, Fatma Kuru, Ezgi Eren Belgin, Serhat Sezgin, Huseyin Cicek, Aziz Bülbül, Gulhan Akbaba, Ayse Gül

**Affiliations:** 1 Department of Energy, Molecular Nano-Materials Laboratory, 52986 Mugla Sıtkı Koçman University, Mugla 48000, Turkiye; 2 Faculty of Health Sciences, 52986 Mugla Sıtkı Koçman University, Mugla 48000, Turkiye; 3 Research Laboratories Center, Immunology and Stem Cell Laboratory, 52986 Mugla Sıtkı Koçman University, Mugla 48000, Turkiye; 4 Faculty of Milas Veterinary Medicine, Muğla Sıtkı Kocman University, Milas, Mugla 48000, Turkiye; 5 Faculty of Medicine, Department of Pathology, 52986 Mugla Sıtkı Koçman University, Mugla 48000, Turkiye; 6 Faculty of Science, Department of Chemistry, 52986 Mugla Sıtkı Koçman University, Mugla 48000, Turkiye; 7 Faculty of Dentistry, 52986 Mugla Sıtkı Koçman University, Mugla 48000, Turkiye; 8 Faculty of Medicine, Department of Endocrinology, 52986 Mugla Sıtkı Koçman University, Mugla 48000, Turkiye

## Abstract

Diabetic foot ulcers are chronic wounds characterized by persistent inflammation and insufficient angiogenesis, leading to delayed healing and substantial clinical burden. This study presents a combined platform in which dental follicle-derived mesenchymal stem cell exosomes (DF-MSC-Exos) are integrated into three-dimensional (3D) electrospun poly­(ε-caprolactone)/gelatin (PCL/GEL) nanofiber scaffolds. The 3D scaffolds—fabricated using a custom collector—exhibited high porosity, rapid wettability, and water-vapor permeability conducive to cell infiltration and a moist wound environment. DF-MSC-Exos (200 μg per 2 × 5 mm scaffold) were loaded onto the nanofibers and evaluated in a streptozotocin-induced diabetic rat foot wound model. Compared with controls and blank scaffolds, exosome-loaded scaffolds accelerated wound closure (reaching 92.5 ± 2.4% by day 21 compared to 61.4 ± 4.0% for control), improved tissue organization, and reduced inflammatory infiltration by H&E analysis. Immunohistochemistry revealed a significant decrease in fibroblast growth factor in the NF + Exos group, a pattern consistent with enhanced early re-epithelialization and tempered late-phase fibroplasia; VEGF exhibited a modest pro-angiogenic increase. These histological and molecular readouts align with a pro-regenerative trajectory—lower leukocytic burden, earlier epithelial coverage, and remodeling compatible with improved scar quality. In summary, DF-MSC-Exos delivered from a 3D PCL/GEL scaffold provide complementary structural guidance and sustained paracrine signaling, yielding faster and qualitatively superior healing in chronic diabetic wounds. This nanofiber–exosome platform is clinically relevant and scalable, and merits further mechanistic and translational evaluation.

## Graphical abstract

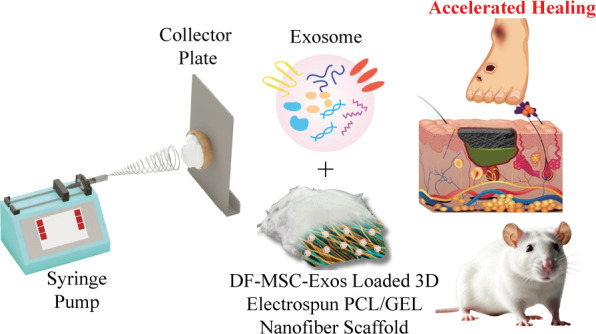

## 1 Introduction

Diabetes mellitus (DM) is a rapidly growing global health problem with increasing incidence and clinical severity, leading to substantial morbidity, mortality, and healthcare costs worldwide. The number of people living with diabetes increased from about 200 million in 1990 to about 830 million in 2022, and diabetes is a leading cause of blindness, kidney failure, cardiovascular disease, and lower-limb amputation worldwide.^1^ Recent estimates from the International Diabetes Federation (IDF) similarly indicate a continuing rise in adult diabetes prevalence, underscoring the growing clinical and health-economic burden.^2^ As DM prevalence rises, chronic and hard-to-heal complications are becoming more common, particularly those driven by vascular dysfunction and impaired tissue repair. Among these, diabetic foot ulcers (DFUs) represent one of the most debilitating outcomes.^3,4^

Diabetic foot ulcers (DFUs) are among the most severe complications of diabetes mellitus, affecting approximately 15% of diabetic patients during their lifetime.^3^ These chronic, full-thickness, nonhealing wounds result from impaired tissue repair processes, including prolonged inflammation, reduced angiogenesis, and disrupted cellular signaling.^4^ DFUs not only diminish the quality of life for patients but also impose significant economic burdens and increase the risks of infection, gangrene, and lower limb amputation.^3–5^ Traditional treatments, such as skin autografts and artificial skin substitutes, are widely used; however, these approaches face limitations such as delayed vascularization, poor integration with healthy tissue, wound contraction, scar formation, and high costs.^6^ Despite advancements in wound care, conventional therapies often fail to achieve effective and complete healing. This failure is largely attributed to core diabetic-wound pathophysiology—persistent chronic inflammation, impaired angiogenesis/perfusion, dysfunctional fibroblast and keratinocyte migration and proliferation, defective extracellular-matrix (ECM) deposition and remodeling, and reduced responsiveness to pro-healing signals—factors that standard dressings and graft-based approaches do not adequately address.^4,6^

Therefore, novel therapeutic strategies are needed to improve clinical outcomes in diabetic wound healing.^5^ While many nanodrug delivery systems (NDDS) have been explored for diabetic wounds, key gaps remain, including insufficient retention of fragile biologics at the wound site, burst release or rapid diffusion, limited protection of therapeutic cargo, and inadequate 3D structural support for deep cellular infiltration and organized tissue remodeling in irregular DFU environments.^6–8^ In parallel, translating biologic therapies such as exosomes to the clinic requires scalable, GMP-compliant manufacturing and rigorous quality control (well-defined identity/purity/sterility criteria and validated potency assays) to mitigate donor- and batch-to-batch variability and to establish appropriate storage and release specifications.^9^ These regulatory considerations further motivate the development of biomaterial carriers that can protect exosomes and enable controlled, localized administration at the wound site.

To address these unmet needs, the integration of biomaterials with cellular therapies and advanced tissue engineering techniques has emerged as a promising innovation in this field.^10^ Among these, mesenchymal stem cells (MSCs) have gained significant attention due to their unique biological properties, including self-renewal, multipotent differentiation potential, and immunomodulatory effects.^11^ MSCs contribute to tissue repair and regeneration through their ability to differentiate into osteoblasts, chondrocytes, and endothelial cells and through the secretion of bioactive molecules and extracellular vesicles (EVs), such as exosomes. These exosomes, measuring 30–150 nm in diameter, facilitate intercellular communication and modulate cellular processes by transporting proteins, growth factors, lipids, mRNAs, and microRNAs to target cells. Recent studies highlight MSC-derived exosomes (MSC-exos) as central mediators of MSCs’ paracrine effects, promoting tissue regeneration, angiogenesis, and immune regulation, making them a promising therapeutic option for DFUs.^12^

Exosomes derived from dental MSCs—including those from dental pulp (DPSCs), periodontal ligament (PDLSCs), and exfoliated deciduous teeth (SHED)—have demonstrated potent pro-angiogenic, anti-inflammatory, and wound-healing properties.^13^ Within this family, dental follicle mesenchymal stem cells (DF-MSCs) represent a particularly advantageous source, as they are ethically unproblematic, derived from discarded tissue during tooth extraction, and possess strong immunomodulatory and regenerative potential. DF-MSC-Exos contain bioactive molecules such as TGF-β, VEGF, HGF, PGE2, and IDO, which suppress inflammation and stimulate fibroblast-mediated collagen and elastin synthesis, thereby enhancing dermal regeneration.^14^ Consistent with this, dental stem cell-derived exosomes have been shown to enhance endothelial responses, promote fibroblast activity and ECM deposition, and modulate macrophage phenotypes toward a pro-healing microenvironment, supporting DF-MSC-Exos as a rational choice for diabetic wound repair.^14,15^

While the therapeutic potential of MSC-derived exosomes is well established, their clinical translation remains limited by challenges in localizing and retaining them at wound sites. Traditionally delivered via subcutaneous injection, exosomes can rapidly diffuse or degrade, leading to suboptimal therapeutic efficacy. In response to these challenges, hydrogel-based delivery systems have been explored, offering improved exosome retention and localized bioactivity. For instance, MSC-exosome-loaded hydrogels have been shown to promote wound closure, angiogenesis, and collagen deposition in diabetic models.^7,8^ However, even these systems may fall short in providing sufficient structural support for cellular infiltration and ECM remodeling in complex wounds. In addition, many hydrogel systems provide limited ECM-like fibrous guidance architecture for coordinated cell migration and tissue organization.^4,16–18^

In contrast, electrospun nanofiber scaffolds offer unique advantages by mimicking the architecture of the ECM, supporting cell adhesion, migration, and proliferation.^16^ These scaffolds also provide a high surface-area-to-volume ratio and interconnected porosity, enabling sustained and localized delivery of bioactive factors while facilitating gas and nutrient exchange and deeper cellular infiltration.^16–18^ Traditional two-dimensional (2D) nanofiber mats, however, limit cell infiltration due to their dense structure. Recent developments in three-dimensional (3D) electrospinning techniques—such as the needle-collector method—enable the fabrication of low-density, compressible, and shape-conforming nanofiber scaffolds suitable for irregular wound geometries.^19,20^ These 3D scaffolds allow for improved tissue integration and sustained bioactive factor delivery, making them ideal for chronic wound healing.^17,18,21^

The choice of the PCL/gelatin (PCL/GEL) matrix is motivated by the need to combine the mechanical stability, biodegradability, and broad biocompatibility of PCL with the hydrophilicity, extracellular-matrix-mimetic character, and cell-adhesive motifs of gelatin, thereby supporting cell attachment and migration while maintaining structural integrity during healing.^22–25^

To the best of our knowledge, this study is the first to investigate the therapeutic synergy between DF-MSC-Exos and 3D electrospun polycaprolactone/gelatin (PCL/GEL) nanofiber scaffolds in a type 1 diabetic foot wound model. While MSC-derived exosomes and nanofiber scaffolds have each been independently studied, their combination—particularly with exosomes from dental follicle MSCs—remains underexplored. By uniting the regenerative bioactivity of DF-MSC-Exos with the structural and biomimetic properties of a cell-permeable 3D scaffold, this approach addresses both biological and mechanical barriers to healing in chronic diabetic wounds.

Therefore, in this study, we fabricated a multifunctional nanofiber scaffold by loading DF-MSC exosomes into a porous 3D PCL/GEL nanofiber. This scaffold can simultaneously inhibit inflammation and promote angiogenesis by releasing DF-MSC exosomes. Furthermore, degradation of the porous nanofiber can promote diabetic skin wound healing by increasing cell migration and collagen deposition (Figure 1). The bioactivity of the scaffold–exosome system was evaluated through in vitro assays and in vivo testing in a type 1 diabetic rat wound model. Outcomes were assessed by measuring wound closure rates, angiogenic response, fibroblast proliferation, and inflammatory modulation. This study introduces a novel, dual-function wound dressing that integrates structural support with sustained biological signaling, offering a promising and clinically translatable solution for DFU treatment.

**Figure 1:**
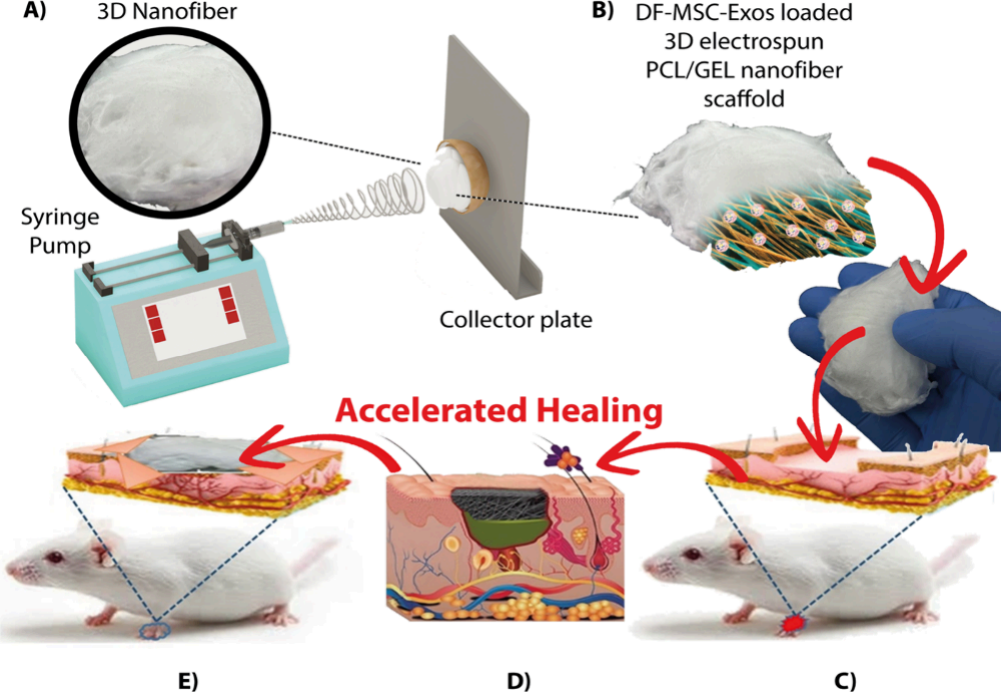
Schematic representation of the preparation of DF-MSC-Exosome-loaded 3D electrospinning-produced PCL/GEL nanofiber scaffolds to support skin wound repair in diabetic rats. (A) Production of 3D nanofibers by electrospinning. (B) Exosome loading and structure of the scaffold. (C) Application of the bioactive scaffold in a rat model (in vivo); histological section. (D, E) Accelerated healing process.

## 2 Materials and Methods

### 2.1 Materials

The materials utilized in this study were obtained from commercial suppliers, and all chemical solvents were of analytical grade, used as received without further purification. Poly­(ε-caprolactone) (PCL, MW ≈ 80,000), gelatin derived from bovine skin Type B (gel strength ≈ 250 g Bloom), trifluoroethanol (TFE), and streptozotocin (STZ) were obtained from Sigma-Aldrich, while the MTT assay kit (Roche, 11465007001, USA) was sourced from Roche. For cell culture, Dulbecco’s Modified Eagle’s Medium (DMEM) (Pan Biotech, P04–01550, Germany), fetal bovine serum (FBS) (Thermo Fisher Scientific, Gibco, A2720801, USA), and penicillin-streptomycin solution (10,000 U/mL) (Pan Biotech, P06–07100, Germany) were used. Exosome isolation was conducted using the ExoQuick ULTRA Exosome Isolation Kit (System Biosciences, EQULTRA-20A-1, USA). To anesthetize the rats for diabetic wound creation, an intramuscular injection of 10 mg/kg xylazine hydrochloride (Rompun, Bayer, 23.32 mg/mL, Germany) and 70 mg/kg ketamine hydrochloride (Ketalar, Parke-Davis, 50 mg/mL, USA) was administered.

### 2.2 Isolation and Characterization of DF-MSCs and DF-MSC-Exos

Human dental follicle (DF) tissues were collected from fully impacted wisdom teeth extracted from five healthy volunteers (aged 19–25 years) with no history of inflammatory or autoimmune diseases. Donors were limited to this young-adult age range (19–25 years) because dental follicle tissue is most consistently available as surgical waste during indicated third-molar extraction, and younger donors typically yield DF-MSCs with higher viability and reduced senescence-related variability, supporting more reproducible exosome production. Nevertheless, donor age may influence MSC phenotype and exosome cargo; this is acknowledged as a limitation and should be evaluated in future studies.

DF-MSCs were isolated following previously established protocols by our team.^26^ Second-passage DF-MSCs were cultured in Dulbecco’s Modified Eagle Medium (DMEM) supplemented with 10% exosome-free fetal bovine serum (FBS) and 1% penicillin/streptomycin until they reached the third passage. Cells were maintained in T75 culture flasks with filter caps at 37 °C in a 5% CO_2_ incubator until 80% confluency. Third-passage cells were trypsinized with 0.25% trypsin-EDTA for 4 min at 37 °C and characterized using flow cytometry.

To identify mesenchymal stem cell (MSC) markers, the cells were stained with PE anti-CD29, FITC anti-CD105, APC anti-CD73, and PerCP anti-CD90 for positive markers, as well as PE anti-CD4, APC anti-CD34, and PerCP anti-CD28 for negative markers. Flow cytometry analysis quantified the marker expression, with results expressed as percentages of fluorescent intensity. The differentiation potential of third-passage DF-MSCs into osteogenic, chondrogenic, and adipogenic lineages was assessed using the StemPro Osteogenesis, Chondrogenesis, and Adipogenesis Kits (Thermo Fisher, USA) under lineage-specific conditions for 14–21 days. At the end of the induction period, staining with Alizarin Red S, Alcian Blue, and Oil Red O confirmed the formation of osteogenic cell colonies with calcium deposits, cartilage with chondrocytes, and adipocytes containing lipid droplets, respectively.

Third-passage DF-MSCs were cultured for 24 h in DMEM supplemented with 10% exosome-free FBS and 1% penicillin/streptomycin. Exosome isolation was performed at the end of the culture period using the ExoQuick ULTRA Exosome Isolation Kit (System Biosciences, EQULTRA-20A-1), following the manufacturer’s protocol. DF-MSC-Exos were successfully isolated using the method as previously described.^27^ The protein content of the isolated exosomes was quantified using the Bicinchoninic Acid (BCA) Protein Assay Kit (Thermo Scientific, A65653, USA), with results reported as μg/mL.^28^

The surface tetraspanins of the isolated exosomes were analyzed by labeling with PerCp anti-CD9 (BD Biosciences, 561329, USA), PE anti-CD63 (BD Biosciences, 557305, USA), and anti-CD81 (BD Biosciences, 551108, USA) antibodies, followed by analysis on a flow cytometer (BD Accuri C6 Plus). Before staining with antibodies, exosomes were incubated with capture beads for flow cytometry analysis. Histogram analysis considered fluorescent intensities of 80% or higher as positive for exosome characterization compared to isotype controls. Additionally, exosomes were examined using field emission scanning electron microscopy (FESEM), and particle size and distribution analyses were performed.

### 2.3 Preparation of 3D Electrospun PCL/GEL Nanofiber Scaffolds

A 10% w/v solution of polycaprolactone (PCL) and a 12% w/v solution of gelatin (GEL) were separately prepared in trifluoroethanol (TFE) at room temperature under magnetic stirring for 24 h. The two polymer solutions were subsequently mixed at a 50:50 weight ratio and stirred for an additional 24 h. The polymer concentrations and the blend ratio were selected based on prior reports demonstrating reliable electrospinnability and a favorable mechanical/hydrophilic balance in PCL/gelatin systems,^22–24^ and were further refined through preliminary optimization to obtain a stable Taylor cone and bead-free fibers. Nanofiber scaffolds were fabricated using a horizontal electrospinning setup (Spingenix, SG100, Palo Alto, US) consisting of a high-voltage DC power supply, a syringe pump, and a 5 mL syringe fitted with a 22-gauge blunt-tip needle. The solution feed rate was maintained at 0.5 mL/h, and the distance between the needle tip and the collector was set to 21 cm, with a voltage of 19 kV applied. The electrospinning parameters (feed rate, tip-to-collector distance, and voltage) were experimentally optimized in preliminary studies to ensure a stable Taylor cone and uniform deposition of 3D, cotton-like nanofiber mats.

3D electrospun scaffolds, a custom-made collector was employed, consisting of an array of 2 cm-long stainless-steel nails attached to a 15 cm-diameter spherical plastic plate to ensure proper electrical grounding, as described in previous studies.^18,21^ A metal nail was positioned at the center of the container, and five evenly spaced metal nail lines were radially arranged from this center point (Figure 2a). Cotton-like 3D electrospun nanofibers were deposited onto the steel nails during the electrospinning process and subsequently collected using a glass rod (Figure 2b). The nanofibers deposited on the spherical collector displayed a three-dimensional, low-density, and uncompressed structure, closely resembling a cotton ball (Figure 2c,d).

**Figure 2:**
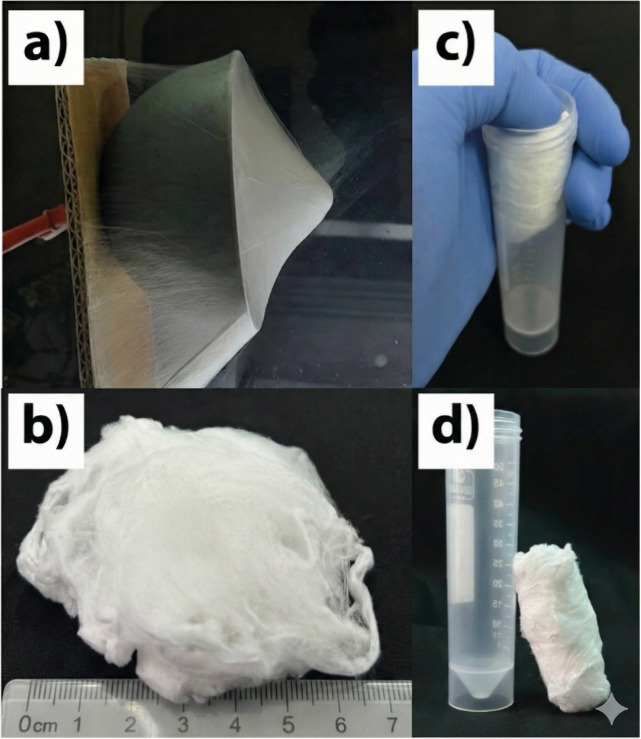
Physical appearance of the 3D PCL/GEL nanofiber scaffolds: (a) Electrospinning process showing nanofiber deposition on the bowl collector, (b) cotton-like 3D PCL/GEL nanofiber mat after removal from the collector, (c) insertion of the nanofiber mat into a sterile tube, and (d) compressibility and shape retention of the 3D PCL/GEL nanofiber scaffold inside the tube.

### 2.4 Characterization of 3D Electrospun PCL/GEL Nanofiber Scaffold

#### 2.4.1 Microstructure Observation

The electrospun scaffolds were sputter-coated with gold for 30 s at a current of 18 mA and subsequently imaged using field emission scanning electron microscopy (FESEM, ZEISS GEMINI-500) at an accelerating voltage of 26 kV. Fiber diameters were determined by randomly selecting approximately 50 fibers from the SEM images and measuring them using ImageJ software (IMAGE J, NIH, Bethesda, MD). Fourier transform infrared (FT-IR) spectroscopy of the scaffolds was performed using a PerkinElmer Spectrum 65 FT-IR spectrophotometer, with spectra recorded in the range of 4000–400 cm^–1^. Thermogravimetric analysis (TGA) was conducted using a PerkinElmer Thermal Analyzer over a temperature range of 30–700 °C at a heating rate of 10 °C/min under a nitrogen atmosphere (2.5 bar, 10 mL/min). In addition, pore size distribution and total porosity were measured by mercury intrusion porosimetry (MIP). Uniaxial tensile and compression stress–strain measurements were performed using a TA Instruments Q800 dynamic mechanical analyzer operated in controlled-force ramp mode (0.1 N/min) in tension and compression at 37 °C (isothermal). Stress–strain curves were used to calculate maximum stress, Young’s modulus and elongation at break (tension) and compressive modulus (compression). Because the objective was to extract quasi-static mechanical parameters, oscillatory DMA (storage modulus, loss modulus and tanδ) was not performed. The water contact angle, indicating the hydrophilic or hydrophobic properties of the scaffold, was measured using a contact angle measuring device (Biolin Scientific Attension Theta Lite). Contact angles were averaged and reported.

#### 2.4.2 Water Vapor Permeability (WVP)

WVP of the nanofiber scaffold was assessed using the container method. Nanofiber scaffold-capped bottles were incubated at 37 °C for 24 h, and the evaporated water was measured. WVP was calculated at steady-state using eq 1.

$$WVP=\frac{W}{AT}$$ 1

where W is the mass of water lost, A is the area of the nanofiber scaffold (1.18 cm^2^), and T is the exposure time.

#### 2.4.3 Water Uptake

The water uptake ratio of the nanofiber scaffolds was determined by cutting samples into 1 cm^2^ pieces, weighing the dry samples (W0), and incubating them in phosphate-buffered saline (PBS, pH 7.4). After 7, 14, and 21 days, the samples were extracted, weighed (W1), and the water uptake was calculated using eq 2.^29^

$$Wateruptake\left(\%\right)=\frac{\left(W1-W0\right)}{W0}\times 100$$ 2

Three measurements were performed for each specimen, and the averages were reported.

#### 2.4.4 Degradation and Biocompatibility

Degradation (weight loss), reported here as scaffold mass loss, was assessed under enzyme-free conditions to establish a reproducible baseline hydrolytic stability of the PCL/GEL matrix and to decouple gelatin dissolution/leaching from enzymatically driven cleavage. Briefly, electrospun scaffolds (n = 3) were dried to constant mass and their initial dry weight (W_0_) was recorded. Samples were then immersed in phosphate-buffered saline (PBS, pH 7.4) at 37 °C under gentle agitation. At predetermined time points, scaffolds were removed, gently rinsed with deionized water to eliminate salts, dried to constant mass, and the remaining dry weight (W_1_) was recorded. Mass loss (%) was calculated using eq 3. Although proteases/collagenase present in chronic wounds can accelerate degradation of gelatin-containing matrices, their activity varies substantially across patients and wound phases; therefore, the enzyme-free PBS condition provides a standardized measure of in vivo-relevant aqueous stability and early phase structural retention.

$$Weightloss\left(\%\right)=\frac{\left(W0-W1\right)}{W0}\times 100$$ 3

In vitro biocompatibility testing of the nanofiber scaffolds was conducted following the protocol described by Schossleitner et al. (2019).^30^ The human keratinocyte cell line found in our cell isolates was suspended in 24-well culture plates with keratinocyte growth medium and planted at 1 × 10^5^ cells/well. The cells were cultured to confluence over 24 h. Subsequently, 1 × 1 cm^2^ nanofiber scaffold was placed on the confluent cell layer, and the cultures were maintained with the growth medium for durations of 24 h, 7 days, and 14 days. At the conclusion of each culture period, cell viability was assessed using Calcein-AM (green) staining for live cells and Ethidium Homodimer-1 (EthD-1, red) staining for apoptotic cells. Fluorescent images were captured using a fluorescence microscope, and the luminescence intensity was compared to a negative control (cells cultured without nanofiber). Viability was quantified by normalizing the luminescence intensity of Calcein-AM staining in the 24 h culture well to 100%. The viability percentages of the 7 day and 14 day cultures were calculated relative to this baseline. All experiments were performed in triplicate to ensure reproducibility.

In vitro cytotoxicity test for the nanofiber scaffolds were conducted based on the methodology described by Lim et al.^31^ The nanofiber scaffolds, with a diameter of 1.6 cm, were washed three times with phosphate-buffered saline (PBS) containing 1% penicillin/streptomycin and subsequently sterilized under ultraviolet (UV) light for 2 h. Human dermal fibroblasts (HDFs) were used for the in vitro culture. Cells (1 × 10^4^) were seeded in 24-well culture plates and cultured in Dulbecco’s Modified Eagle Medium (DMEM) supplemented with 10% fetal bovine serum (FBS) and 1% penicillin-streptomycin in a CO_2_ incubator maintained at 37 °C and 5% CO_2_. Once the cells reached 80% confluence, they were cultured in the presence or absence of the nanofiber scaffolds for 72 h. For cell cytotoxicity tests, 1 × 10 ^4^ HDF cells were seeded on the 1.6 cm (diameter) nanofiber scaffolds in 24-well plates with DMEM medium and cultured for 3 days. At the end of the culture period, DMEM medium was discarded and fresh DMEM medium was added. Fresh DMEM with MTT solution (5 μg/L) was added to each well and incubated for 4 h at 37 °C. The medium was discarded, and 1 μL of DMSO was added to dissolve the formed formazan. After dissolving the formazan crystals, 100 μL aliquots were transferred to a 96-well culture plate with five replicates per sample. Absorbance was measured at a wavelength of 570 nm using a microplate spectrophotometer. The absorbance data obtained at the end of the measurement were compared with the other wells, considering the control well as having 100% viability.

The cultures were incubated for 4 h at 37 °C to allow formazan crystals to form. After incubation, the medium was discarded, and 200 μL of dimethyl sulfoxide (DMSO) was added to dissolve the formazan crystals. Aliquots of the dissolved formazan were transferred to a 96-well culture plate, with five replicates per sample. Absorbance was measured at a wavelength of 570 nm using a microplate reader. The absorbance values were compared across wells, with the control well (no nanofiber) set as 100% viability. The results were recorded as viability percentages (%).

### 2.5 Preparation of Exosome-Loaded 3D Electrospun PCL/GEL Nanofiber Scaffolds and In Vitro Exosome Release Assay

Electrospun 3D PCL/GEL nanofiber scaffolds were cut into 2 mm × 5 mm rectangles and sterilized under UV light (30 min per side) prior to exosome loading. For in vivo application, the target exosome dose was selected based on prior reports demonstrating efficacy in comparable diabetic wound models, where ∼200 μg exosomes per treatment is commonly used.^32^ Exosome loading was performed by passive adsorption. DF-MSC-derived exosomes were suspended in sterile PBS, and 200 μg of exosomal protein (as quantified by the BCA assay) was prepared in a small loading volume (50 μL) for each scaffold. The suspension was slowly pipetted dropwise onto the scaffold surface, allowed to absorb, and incubated at 4 °C for 2 h to promote passive adsorption of exosomes onto/into the porous nanofiber network. After incubation, scaffolds were used immediately for in vivo application or transferred to release studies. When short-term storage was required, exosome-loaded scaffolds were kept in sterile sealed tubes at 4 °C, protected from light, and used within 24 h; prolonged storage and freeze–thaw cycles were avoided to minimize potential loss of exosome bioactivity.

For the in vitro exosome release assay, each exosome-loaded scaffold was placed in an individual well of a 24-well plate containing 200 μL PBS (37 °C, gentle shaking). At predetermined time points, the entire supernatant was collected and replaced with fresh PBS to maintain sink conditions. Released exosomes were quantified using total protein content (BCA) as a surrogate of cumulative release, and the day-21 released fraction was further characterized for exosome surface markers, as reported in the Results (Tables 1 and 2). This protocol was used to assess scaffold-associated retention and sustained release behavior.

**Table 1:** Total Protein Content of DF-MSC-Exos

days	PCL/GEL (μg/mL)	DF-MSC-Exos (μg/mL)	PCL/GEL + DF-MSC-Exos (μg/mL)
0	0.0 ± 0.0	194.0 ± 5.0	5.0 ± 2.0
7	0.0 ± 0.0	198.0 ± 4.0	53.0 ± 3.0
14	0.0 ± 0.0	195.0 ± 2.0	132.0 ± 6.0
21	0.0 ± 0.0	197.0 ± 5.0	186.0 ± 2.0

**Table 2:** Exosome Positive Markers on Day 21

positive markers of exosomes (%)			
CD9	CD63	CD81	HSP90
69.2	60.4	63.1	73.4

### 2.6 Construction of Diabetic Rat Full-Thickness Foot Skin Wound Model

Ethical approval was obtained from the Institutional Ethics Committee of Muğla Sıtkı Koçman University (Approval No. 23/21). Human dental follicle tissues (donors aged 19–24 years undergoing indicated third-molar extraction) were collected after written informed consent and in accordance with institutional ethical standards. The study was conducted and reported in accordance with the ARRIVE guidelines, and all procedures were carried out in accordance with relevant national and institutional guidelines and regulations.

6–8 week-old male Wistar albino rats were used to create diabetic rat foot skin wound models. The animals were housed at a controlled room temperature of 25 °C with a 12 h light–dark cycle and provided with a standard diet and tap water. Diabetes was induced by a single intraperitoneal injection of streptozotocin (STZ) at a dose of 55 mg/kg in citrate buffer (pH 4.4, 0.1 M). Age-matched control rats received an equivalent volume of citrate buffer via intraperitoneal injection. After 24 h, blood glucose levels were measured using a glucometer from blood samples collected from the tail vein. Blood glucose levels exceeding 300 mg/dL confirmed the successful induction of diabetes.^33^ Diabetic rats were anesthetized via intraperitoneal injection of ketamine (100 mg/kg) and xylazine (5 mg/kg). One foot was shaved and disinfected with povidone–iodine. Using a flexible transparent template, a 2 × 5 mm rectangle was outlined on the dorsal surface of that foot, and a full-thickness excisional wound was created by removing the marked skin.

The diabetic full-thickness wound was treated with gauze (Control), 3D electrospun PCL/GEL nanofiber scaffold (NF) and 3D PCL/GEL nanofiber scaffolds loaded with DF-MSC-Exos (NF-Exos) sequentially and then wrapped with gauze for immobilization. On days 7, 14 and 21, measurements were taken from the wound area to allow macroscopic examination of wound healing and observation of the gross effect. The wound healing was recorded with a camera and analyzed with ImageJ software.

$$Wound\;healing\;rate(\%)=((A_{0}-A_{t})/A_{0})\times 100$$ 4

A_0_ is the original area of the trauma, and A_t_ is the area of the trauma at the point time.

### 2.7 Immunohistochemical and Pathological Analyses

Twenty-one days after surgery, the animals were euthanized via ketamine overdose injection, and tissue samples were collected for analysis. Half of the samples were frozen, while the remaining were fixed in 10% formalin, processed, embedded in paraffin, sectioned, and stained with hematoxylin and eosin (H&E). The prepared slides were examined and interpreted under a light microscope (Carl Zeiss, Thornwood, USA) equipped with a digital camera (Olympus, Tokyo, Japan), and images were captured at ×100 and ×200 magnifications.

Comparative evaluations of ulcer presence and inflammatory cell infiltration were conducted across the groups. Ulcer presence was scored as 1 for the presence of ulcers and 0 for the absence of ulcers. Inflammatory cell infiltration was scored on a scale from 0 to 3, where 0 indicated no inflammatory cells, 1 represented weak infiltration, 2 represented moderate infiltration, and 3 indicated severe infiltration. Protein expression levels were also assessed and scored based on intensity, with 0 indicating no expression, 1 indicating weak expression, 2 indicating moderate expression, and 3 indicating severe expression. These analyses provided detailed insights into ulceration, inflammatory responses, and protein expression levels among the study groups.

### 2.8 Statistical Analysis

Statistical analyses were performed using the GraphPad Prism 8.0 software (GraphPad Software, Inc., CA, USA). Data were expressed as mean ± standard deviation (SD). Comparisons among more than two groups were conducted using one-way analysis of variance (ANOVA), while unpaired Student’s *t* tests were used for comparisons between two groups. A p-value of less than 0.05 (*p* < 0.05) was considered statistically significant.

## 3 Results and Discussion

### 3.1 Characterization of DF-MSCs and DF-MSC-Exos

Dental follicle mesenchymal stem cells (DF-MSCs) represent a promising source for regenerative applications due to their accessibility from routine dental procedures and robust biological properties. In this study, DF-MSCs were isolated from human dental follicles and characterized to confirm their mesenchymal stem cell identity. The isolated cells exhibited the typical fibroblast-like morphology and colony-forming capacity associated with MSCs (Figure S1). The cells successfully differentiated into osteogenic, chondrogenic, and adipogenic lineages (Figure S2). Flow cytometry analysis of third-passage cells revealed high expression of DF-MSCs-positive markers (CD29:98.9%, CD105:99.2%, CD73:95.2%, CD90:97.6%) and minimal expression of DF-MSCs-negative markers (CD14:1.7%, CD34:0.6%, CD28:1.2%) (Figure S3).

Isolated DF-MSC-Exos expressed the positive surface markers CD9, CD63, and CD81, as confirmed by flow cytometry analysis with strong luminescence intensity (Figure S4a). As observed in the FESEM images (Figure S4b), the isolated exosomes displayed dimensions ranging from 30 to 150 nm, consistent with international exosome standards.^34,35^ TRPS analysis (Figure S4c) showed a histogram with a single peak around 90 nm, corresponding to the mean diameter. Nanoparticle size distribution analysis further confirmed that 97.2% of the particles fell within the 30–150 nm range. These results are in line with some previously reported morphology and size distribution of the Exos.^36–38^

### 3.2 Characterization of 3D PCL/GEL Nanofiber Scaffold

The 3D PCL/GEL nanofiber scaffolds fabricated using a custom electrospinning setup formed a cotton-like, highly porous three-dimensional mat (Figure 3a). Here, the terms “cotton-like” and “low-density” describe the macroscopic, uncompressed appearance of the scaffold (Figure 2) and are supported by FESEM observations. Representative FESEM micrographs, fiber diameter distribution, and porosity analysis are shown in Figure 3b–d. The images reveal a randomly oriented 3D fibrous network, which may provide a favorable microenvironment for cell attachment and infiltration. Quantitatively, the average fiber diameter was 1300 ± 48 nm and the image-based scaffold porosity was 76% (Figure 3c,d). In addition, mercury intrusion porosimetry (MIP) indicated an average pore (throat) diameter of ∼11.8 μm and a total porosity of ∼70.2% for the 3D PCL/GEL scaffold. The small difference between the FESEM-derived (image-based) and MIP-derived porosity values is expected due to method-dependent definitions (2D area fraction vs 3D intrusion-based metrics) and the pressure-based nature of MIP measurements on soft fibrous mats. Smaller fiber diameters are generally associated with reduced pore size in nanofibrous scaffolds.^39^ Porosity is a key parameter for wound healing and tissue engineering applications because it supports cellular infiltration and mass transport; an optimal porosity range of 60–90% has been reported for tissue engineering scaffolds.^40^ The smooth fiber surface and relatively uniform fiber diameter distribution further support the suitability of the 3D PCL/GEL scaffold for tissue engineering and wound healing applications. Mat thickness/bulk density was not quantified as an independent parameter because the 3D structure is highly compressible.

**Figure 3:**
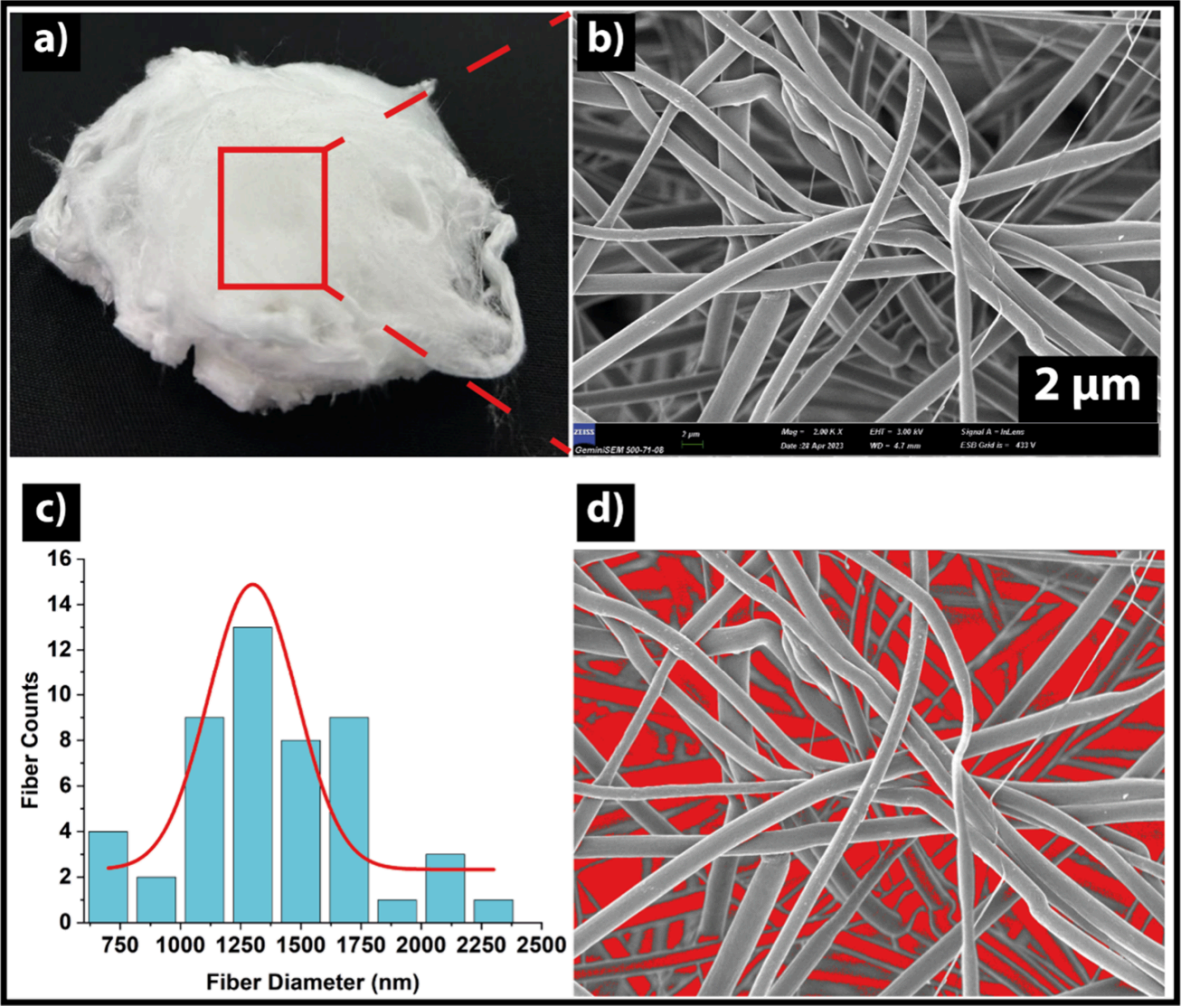
(a) Image of 3D PCL/GEL nanofiber scaffold. (b) FESEM image. (c) Fiber diameter distributions. (d) Porosity for 3D PCL/GEL nanofiber scaffolds.

Electrospun nanofibrous scaffolds are particularly suitable for wound healing because their ECM-mimicking fibrous architecture and high surface area provide abundant sites for cell attachment and contact guidance, supporting cell spreading and migration. Their interconnected porous network facilitates oxygen diffusion and mass transport while absorbing wound exudate, helping to maintain a moist microenvironment that favors re-epithelialization. In addition, the breathable fibrous mat can act as a protective barrier against external contaminants while preserving moisture balance, which is desirable for advanced wound dressings.^41^

Beyond these structural functions, electrospun matrices can be engineered as local delivery platforms for bioactive agents. By tuning polymer composition and fiber architecture, the scaffold can improve the retention of exosomes at the wound site and enable more sustained release, reducing rapid wash-out and prolonging exposure during critical phases of repair. Sustained presentation of exosome cargo may support pro-regenerative signaling, including angiogenesis and granulation tissue formation.^41^

The FTIR analysis confirmed the molecular structures of PCL, GEL, and their composite scaffold (Figure S5). For PCL, characteristic peaks at 2941 cm^–1^ and 2866 cm^–1^ corresponded to CH_2_ and C = O stretching vibrations, with a strong carbonyl peak at 1722 cm^–1^ and C–O–C stretching at 1287 cm^–1^, 1240 cm^–1^, and 1166 cm^–1^, consistent with literature.^42,43^ For GEL, amide I and II peaks were observed at 1632 cm^–1^ and 1527 cm^–1^, alongside C–H and N–H bending vibrations at 1447 cm^–1^, 1334 cm^–1^, and 1234 cm^–1^.^22^ The PCL/GEL composite exhibited overlapping peaks, confirming successful blending. Key peaks included CH_2_ stretching at 2933 cm^–1^, C = O stretching at 2864 cm^–1^, and ester and amide contributions at 1720 cm^–1^, 1638 cm^–1^, and 1527 cm^–1^.^23^ These results demonstrate structural integrity and compatibility for biomedical applications, aligning with previous studies.^24^

The TGA thermogram for PCL, GEL, and the PCL/GEL composite scaffold (Figure S6) reveals the characteristic thermal degradation patterns of the materials. PCL shows single-step degradation with stability up to ∼400 °C and a sharp weight loss between 400 and 450 °C. GEL remains stable up to ∼200 °C, with degradation occurring between 300 and 500 °C, typical of gelatin’s polypeptide backbone decomposition.^44^ The PCL/GEL composite exhibits a degradation profile influenced by both components, with weight loss between 300 and 450 °C, indicating interaction between PCL and GEL that modifies the thermal behavior. The composite’s enhanced stability at lower temperatures compared to pure GEL is due to PCL’s stabilizing effect within the matrix.^45^

The dynamic mechanical analysis of PCL/GEL nanofiber scaffolds; tensile stress–strain and compressive stress–strain curves are shown in Figures S7 and S8, respectively. The tensile strength was determined to be 1.151 MPa, with an elongation at break of 18.93% and a Young’s modulus of 0.03499 MPa. The compressive strength and modulus were measured at 0.2548 and 0.073 MPa, respectively. These mechanical properties suggest that the PCL/Gel nanofibrous scaffold possesses adequate flexibility and mechanical compatibility, making it well-suited for soft tissue engineering applications.^46^

Surface contact angle measurements of PCL/GEL nanofiber scaffolds, shown in Figure S9, reveal their highly hydrophilic nature, with contact angles dropping to nearly zero within 5 s. While PCL is inherently hydrophobic, the incorporation of GEL significantly enhances the scaffolds’ hydrophilicity,^25^ resulting in a zero-contact angle. This property makes the PCL/GEL scaffolds highly suitable for absorbing wound exudates and maintaining a moist wound bed. Effective wound dressings must meet these criteria, as surface wettability and hydrophilicity play a crucial role in supporting cell adhesion and growth.^47^

Water Vapor Permeability (WVP) of the PCL/GEL nanofiber scaffold was measured as 219.26 g/m^2^·h, falling within the recommended range of 200–500 g/m^2^·h for wound dressings.^48^ This range ensures adequate moisture regulation, preventing fluid accumulation while avoiding excessive dehydration. Vapor exchange is a critical factor in determining the efficacy of wound dressings, as high WVP can lead to dehydration and scar formation, whereas low WVP can delay healing due to retained exudates.^49^ Thus, the measured WVP value indicates that the PCL/GEL scaffold exhibits an optimal balance, suitable for effective wound healing.

Water uptake ratio of the PCL/GEL nanofiber scaffold was 1060%, as shown in Figure S10, attributed to the hydrophilic properties of GEL. After reaching this maximum, water retention rates declined, aligning with biodegradation test results. This decrease is linked to the opening of polymer chains during scaffold degradation, leading to the reverse leakage of retained liquid. These findings confirm that the PCL/GEL scaffold effectively absorbs wound exudates, supporting its role in enhancing the wound healing process.

In vitro degradation/mass loss of the PCL/GEL nanofiber scaffold, shown in Figure S11, indicates approximately 60% mass loss within the first 7 days and ∼90% by day 21. This test was performed in enzyme-free PBS to provide a baseline hydrolytic/leaching profile under physiological ionic strength without introducing enzyme-specific variability. Because PCL is known to degrade slowly under purely hydrolytic conditions,^50,51^ the rapid early mass loss is most plausibly attributed to gelatin dissolution/leaching^52^ and subsequent structural disintegration of the composite rather than accelerated chemical degradation of the PCL component. Therefore, the reported values should be interpreted as composite mass loss. In vivo, proteases/esterases and cellular activity may further accelerate degradation; nevertheless, the scaffold remained functional throughout the 21 day diabetic wound study, supporting adequate stability for the intended healing window. Future work will include enzyme-assisted degradation tests (e.g., collagenase/protease and lipase)^50^ and polymer molecular weight analysis to decouple gelatin leaching from true PCL chain degradation.

### 3.3 Biocompatibility and Cytotoxicity of PCL/GEL Nanofiber

It is important that the wound dressing is biocompatible and does not cause toxicity to the cells in the wound area.^53^ Biocompatibility test was conducted using human keratinocytes. The viability of control cells at 24 h, 7 days, and 14 days was 100.0 ± 0.0%, 98.1 ± 0.5%, and 92.3 ± 0.8%, respectively. The presence of PCL/GEL nanofibers did not significantly affect cell viability within the first 24 h (97.3 ± 0.6%). At the end of 7 days and 14 days, cell viability in the PCL/GEL nanofiber group was 94.82 ± 0.64% and 90.26 ± 0.48%, respectively (Figure S12). Additionally, the MTT assay demonstrated 92.78 ± 1.22% viable cells compared to control cultures, further supporting the biocompatibility of the PCL/GEL nanofibers.

### 3.4 Total Protein Content and Release Profile of DF-MSC-Exos

Before the in vivo experiments, exosome loading, release, and retention in the PCL/GEL scaffolds were evaluated as described in a previous study with slight modifications.^38^ Total protein content of DF-MSC-Exos, measured either as free exosomes (input) or as exosomes released from the PCL/GEL scaffolds into PBS, was quantified by BCA assay. Release from exosome-loaded scaffolds was monitored over 21 days. Control scaffolds without exosomes exhibited minimal background protein, confirming that the measured signal predominantly reflected exosome-derived protein. Cumulative analysis showed that >95% of the loaded exosomes were released by day 21. Detailed quantitative data are provided in Table 1.

On day 21, the released exosomes were further analyzed via flow cytometry for the expression of positive surface markers. The flow cytometry data are provided in Table 2.

### 3.5 Evaluation of In Vivo Wound Closure in Diabetic Rats

To evaluate the wound-repair efficacy of exosome-loaded nanofibers, we established a diabetic rat foot-skin wound model and monitored the healing of 3D PCL/GEL nanofiber scaffolds loaded with DF-MSC-Exos. Wound dimensions were recorded on postoperative days 0, 7, 14, and 21 (Figure 4a,b). By day 7, healing was evident, initiating at the wound edges and progressing toward the center; during this phase, a discernible eschar formed over the wound surface. As shown Figure 4c, wound healing rates were 22.0 ± 2.1% (control), 31.8 ± 3.5% (NF), and 44.2 ± 3.9% (NF + Exos). On day 14, scabs had peeled off and wound areas had further decreased in all groups except the control group; the corresponding healing rates were 45.3 ± 3.6% (control), 63.7 ± 3.8% (NF), and 83.6 ± 3.1% (NF + Exos). By day 21, wounds continued to contract, with healing rates of 61.4 ± 4.0% (control), 86.0 ± 3.2% (NF), and 92.5 ± 2.4% (NF + Exos). Collectively, these results indicate that NF-Exos scaffolds most effectively promote tissue repair in diabetic wounds. The NF + Exos treatment showed the fastest diabetic wound healing, leaving almost no wound area on day 21, as detailed in the average wound area measurements provided in Table 3 (absolute wound area values in mm^2^), complementing Figure 4 which presents the wound-closure trajectory and percentage closure. The skin area covered with newborn hair on day 21 showed that the hair-covered area reached 85% of the wounds in the NF+ Exos group and 76% in the NF group. This suggests that DF-MSCs-loaded Nanofiber can promote new hair growth on the skin.

**Table 3:** Absolute Wound Area Values (mm^2^) at Days 0, 7, 14, and 21 Used to Calculate Percentage Wound Closure Shown in Figure 4c

groups	day 0	day 7	day 14	day 21
control	14.04 ± 0.6	10.95 ± 0.5	7.67 ± 0.7	5.41 ± 0.8
NF	16.24 ± 0.4	11.08 ± 0.6	5.90 ± 0.4	2.27 ± 0.9
NF + Exos	15.08 ± 0.4	8.41 ± 0.5	2.47 ± 1.2	1.13 ± 0.9
P value	*P* < 0.05	*P* < 0.05	*P* < 0.05	*P* < 0.05

**Figure 4:**
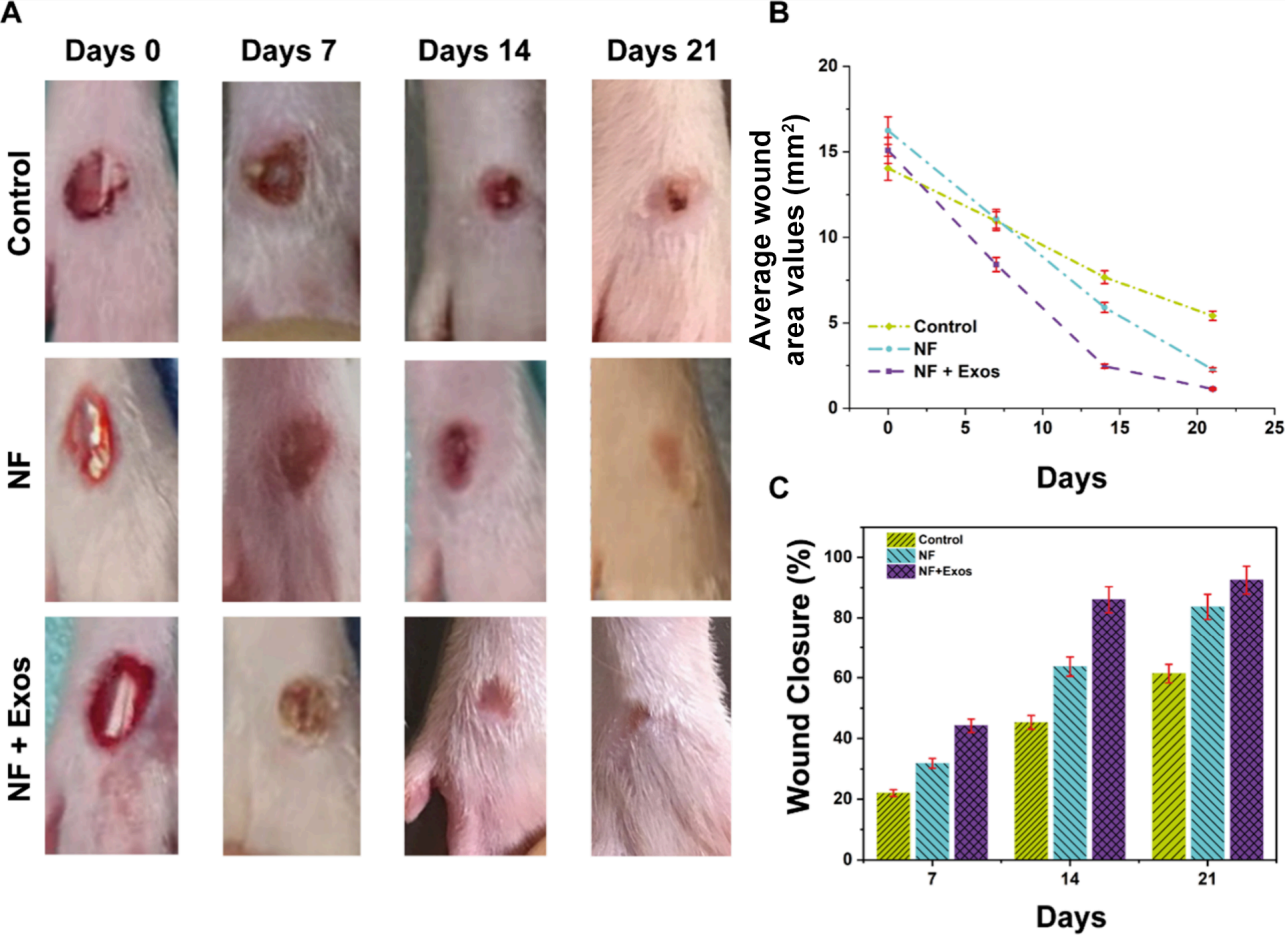
Control, NF, and NF + Exos groups: (A) Representative photographs showing wound closure on days 0, 7, 14, and 21. (B) Wound area reduction (mm^2^) over the 21 day treatment period. (C) Percentage wound closure at the indicated time points (mean ± SD, *n* = 6). Differences among groups at each time point were evaluated using one-way ANOVA (*p* < 0.05).

### 3.6 Histopathological Evaluation

To assess the impact of DF-MSC-Exos–loaded PCL/GEL nanofiber scaffolds on diabetic foot wound repair, we evaluated ulceration, inflammatory cell infiltration, and histological architecture by H&E staining (Figure 5a). Consistent with a pro-regenerative shift, inflammatory cell density appeared lowest in NF + Exos (0.4 ± 0.1), followed by NF (0.8 ± 0.2) and Control (1.5 ± 0.3). Similarly, ulceration scores were lowest in NF + Exos (0.1 ± 0.1) and NF (0.2 ± 0.1) compared with Control (0.5 ± 0.1). One-way ANOVA indicated a significant difference among groups (*p* < 0.05) (Figure 5b). These histological improvements align with the recognized immunomodulatory actions of MSC-derived exosomes—particularly their capacity to dampen excessive inflammation via macrophage polarization toward an M2 phenotype in hyperglycemic wounds.^54^ The scaffold context likely contributed as well: electrospun PCL/gelatin dressings provide a moist, cell-adhesive microenvironment and have been shown in diabetic rodent models to support organized granulation while limiting inflammatory burden.^55^ Taken together, the reduced leukocytic infiltration and lower ulceration observed in the NF + Exos group support a combined regenerative (re-epithelialization/granulation) and immunomodulatory (inflammation attenuation) effect, consistent with established EV-based mechanisms in skin repair.^56^

**Figure 5:**
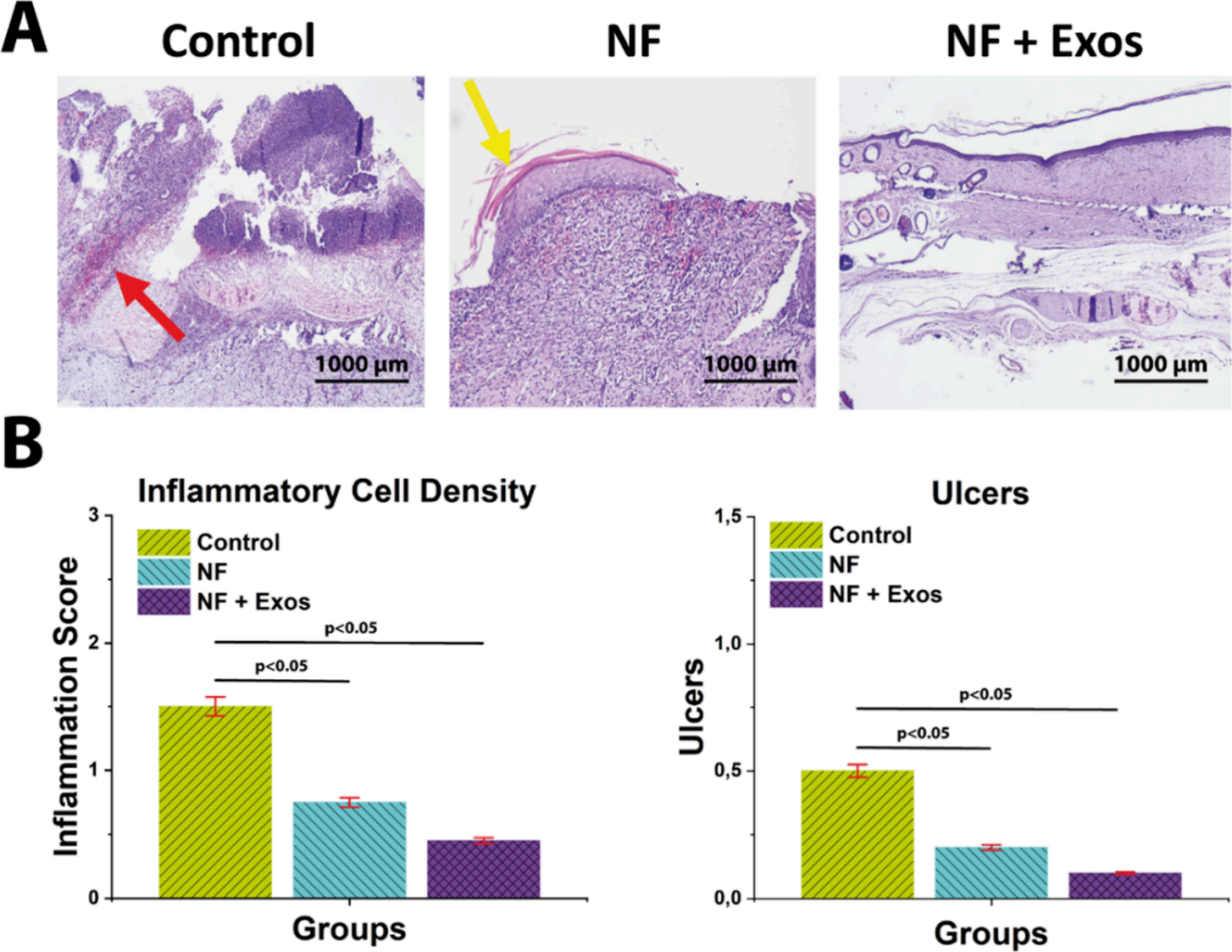
H&E-stained images and statistical analysis of ulcers and inflammation. (A) Representative light microscope images show inflammatory cells (red arrows) and ulcers (yellow arrows). (B) Semiquantitative scores (mean ± SD, *n* = 6) for inflammatory cell density and ulceration on day 21. One-way ANOVA was used to evaluate differences among groups (*p* < 0.05 for overall group effect).

DF-MSCs are known to exert paracrine effects that can amplify host-tissue signaling, including the up-regulation of key wound-healing growth factors such as EGF, FGF and VEGF; this is consistent with broader evidence that MSC-derived exosomes reprogram the wound microenvironment toward regeneration.^15^ In diabetic foot wounds—where repair is delayed—we profiled FGF (fibroblast proliferation and scar formation), EGF (early re-epithelialization) and VEGF (angiogenesis), three axes that collectively orchestrate proliferation and remodeling.^57^

As shown in Figure 6, FGF expression was lowest in NF + Exos (0.3 ± 0.1), followed by NF (0.7 ± 0.2) and Control (1.9 ± 0.3), with a significant difference among groups (*p* < 0.05). Conversely, EGF expression was highest in NF (1.2 ± 0.2) relative to NF + Exos (0.6 ± 0.1) and Control (0.1 ± 0.1), and the difference among groups was significant (*p* < 0.05). VEGF showed a modest increase in NF (0.6 ± 0.1) versus the other groups (0.0 ± 0.0). The corresponding protein expression patterns were visualized through immunohistochemical staining as shown in Figure 7. Interpreted in a temporal framework, the late-phase reduction of FGF aligns with a transition from proliferative granulation to remodeling: FGF-2 supports early fibroblast expansion and provisional matrix deposition, but sustained elevation can drive excessive fibroplasia and disorganized collagen; attenuation in the NF + Exos group therefore suggests narrowed fibrotic signaling and improved matrix remodeling, a pattern associated with better scar quality.^57^

**Figure 6:**
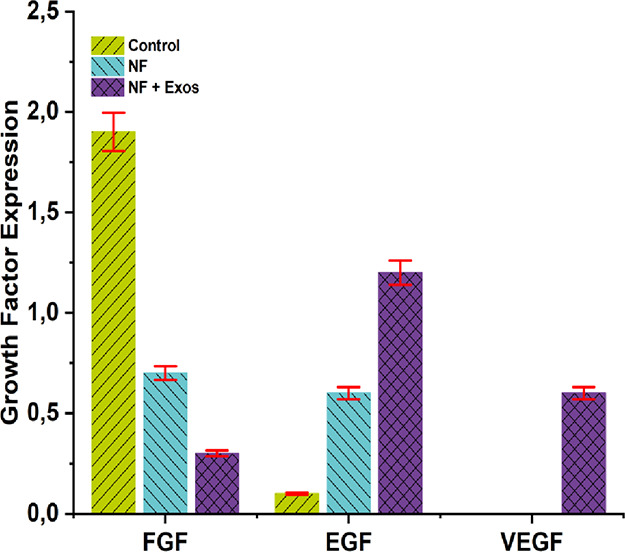
Semiquantitative analysis of growth factor expression in wound tissue on day 21. Immunohistochemical scoring (0–3; see Materials and Methods) is shown for (i) FGF, (ii) EGF, and (iii) VEGF in the Control, NF, and NF + Exos groups. Data are presented as mean ± SD (*n* = 6). One-way ANOVA indicated a significant overall difference among groups (*p* < 0.05).

**Figure 7:**
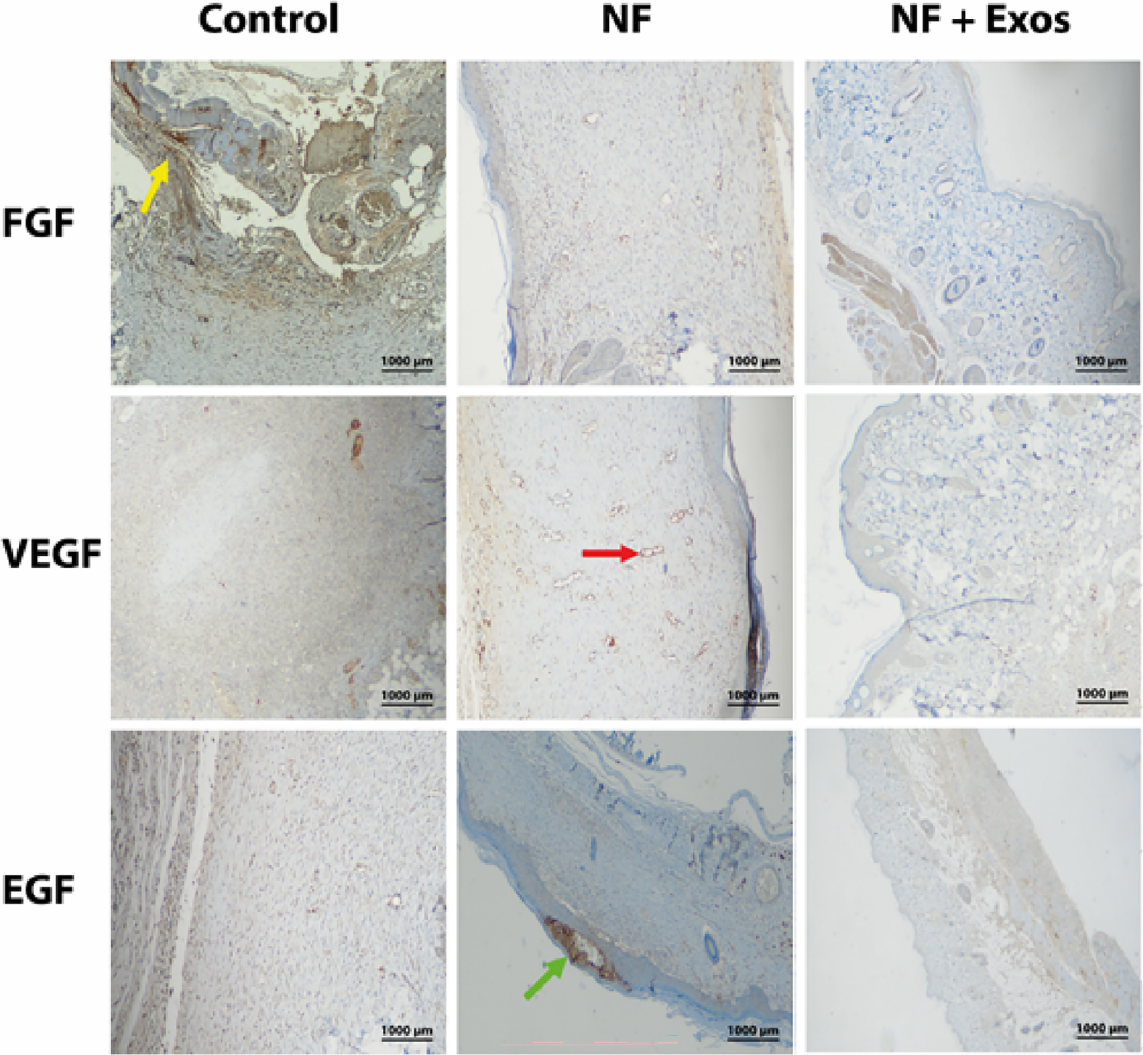
Representative immunohistochemical images showing positive staining for growth factors (FGF, VEGF, and EGF) in wound tissue sections on day 21 in the Control, NF, and NF + Exos groups. FGF-positive staining is indicated by yellow arrows, VEGF-positive staining by red arrows, and EGF-positive staining by green arrows. Scale bar: 1000 μm.

Concurrently, the EGF increase is consistent with accelerated keratinocyte migration and proliferation that underpins rapid re-epithelialization; clinical-preclinical syntheses continue to position the EGF/EGFR axis as a proximal coordinator of epithelial repair.^58^ EGF expression shows early re-epithelization of the tissue. The upward trend in VEGF is compatible with a pro-angiogenic milieu during the proliferative phase, in which neovessel formation supports granulation tissue and subsequent tissue maturation.^59^

Taken together—EGF and VEGF increases with concomitant late-phase FGF down-regulation—our findings indicate that NF shifts the wound microenvironment toward a pro-regenerative trajectory: earlier closure through epithelialization and angiogenesis, followed by restrained fibroplasia during remodeling. In addition, NF + Exos completed re-epithelization and regeneration earlier than NF, therefore the growth factors were slightly expressed in the tissue sections. This pattern mirrors the established phase-specific roles of these growth factors and the documented capacity of MSC-derived exosomes to modulate them in chronic wounds.^60^

Several recent studies have explored extracellular vesicle (EV)/exosome-based approaches for diabetic or full-thickness skin wound healing. Most of these strategies rely on hydrogel carriers to improve local retention and preserve bioactivity. In this context, hydrogels loaded with MSC- or platelet-derived exosomes have been shown to accelerate wound closure, enhance collagen deposition, and stimulate angiogenesis in diabetic wound models.^7,8,12,37^ In parallel, electrospun nanofiber dressings have attracted broad interest because their ECM-mimicking architecture, high surface area, and interconnected porosity can facilitate cell attachment and migration while supporting exudate management and gas/moisture exchange. Among these, PCL/gelatin blends are particularly appealing, as they combine the mechanical robustness of PCL with the hydrophilicity and cell-adhesive motifs of gelatin, and have demonstrated good cytocompatibility in skin-related applications.^16–18,20,21,31^ More recently, hybrid platforms that integrate electrospun fibers with exosome delivery have been proposed to unite structural guidance with sustained paracrine signaling. For instance, exosome-loaded alginate/PCL fiber composites and related fiber-based delivery systems have been reported to improve re-epithelialization and vascularization in full-thickness wound models, underscoring the value of a fibrous matrix for localized and prolonged EV presentation.^36–38^ Compared with these approaches, our platform integrates DF-MSC-derived exosomes with a low-density, compressible 3D PCL/GEL nanofiber mat, enabling conformal placement in a small foot wound geometry and providing a cell-permeable 3D microenvironment. The 3D architecture used here (uncompressed, highly porous) differs from conventional dense 2D mats that can restrict infiltration; similar low-density 3D electrospun constructs have been shown to promote cell ingrowth.^18,21^ In the STZ-induced diabetic rat foot wound model, DF-MSC-Exos delivery from the 3D scaffold resulted in faster closure and improved histological quality relative to blank scaffolds and gauze controls, supporting the concept that combining a 3D fibrous dressing with exosome signaling can address both structural and biological barriers in chronic diabetic wounds.

Because dressing–wound conformity and retention are critical determinants of therapeutic performance—particularly for small, irregular wound geometries—future designs could further optimize placement and reduce postapplication displacement. Along these lines, deployable nanofibrous matrices based on shape-memory polymer (SMP) concepts have been proposed. In such systems, external triggers (e.g., near-infrared (NIR) light) can induce controlled shape recovery, facilitating deployment and conformal contact on irregular tissue surfaces. In this area, Vilay et al. reported NIR-responsive shape-memory composite nanofibers as deployable matrices for biomedical applications.^61^ Although the present PCL/GEL scaffold is not an SMP construct, incorporating stimuli-responsive, self-deploying fiber architectures represents an attractive future direction to further improve placement stability and retention of exosome-delivering wound dressings.

This study primarily aimed to establish in vitro characterization and demonstrate in vivo proof-of-concept efficacy; therefore, several postmanufacturing considerations relevant to clinical translation were not systematically addressed. While scaffolds were handled under sterile conditions and UV sterilization was used for laboratory experiments, clinically compatible terminal sterilization methods (e.g., gamma irradiation or ethylene oxide) were not evaluated, and their potential effects on fiber morphology and exosome integrity should be investigated in future work.^9,20^ In addition, formal stability testing was not performed for either neat or exosome-loaded scaffolds. Neat mats were stored dry in sterile, sealed tubes protected from light and moisture, and exosome-loaded scaffolds were used immediately after loading or kept only briefly at 4 °C prior to use. However, no accelerated or long-term studies were conducted to assess poststorage microstructure and mechanical performance of the mats, or exosome bioactivity/potency after incorporation. Future studies should incorporate validated sterile packaging, define GMP-aligned storage conditions (including temperature, humidity/light protection, and duration), and implement stability and potency assays to establish shelf life, reproducibility, and overall translational readiness.^9^

## Conclusions

This study demonstrates that integrating dental follicle-derived mesenchymal stem cell exosomes (DF-MSC-Exos) into a three-dimensional (3D) electrospun PCL/GEL scaffold yields synergistic benefits for diabetic foot wound repair in a type-1 diabetic rat model. In vivo, the NF + Exos dressing achieved markedly faster wound closure, with healing rates of 44.2 ± 3.9% (day 7), 83.6 ± 3.1% (day 14), and 92.5 ± 2.4% (day 21), compared with blank nanofibers (31.8 ± 3.5%, 63.7 ± 3.8%, and 86.0 ± 3.2%, respectively) and control dressings (22.0 ± 2.1%, 45.3 ± 3.6%, and 61.4 ± 4.0%), corresponding to a + 31.1 percentage-point improvement versus controls at day 21. Hair regrowth at day 21 further supported improved skin restoration, reaching 85% coverage in NF + Exos versus 76% in NF (Figure 4). Histopathology confirmed reduced inflammation and improved tissue quality: inflammatory cell density decreased to 0.4 ± 0.1 in NF + Exos versus 0.8 ± 0.2 in NF and 1.5 ± 0.3 in controls (*p* < 0.05), alongside lower ulceration scores (0.1 ± 0.1 vs 0.2 ± 0.1 and 0.5 ± 0.1) (Figure 5). In vitro, the platform showed sustained exosome-associated protein release (53.0 ± 3.0 μg/mL at day 7; 132.0 ± 6.0 μg/mL at day 14; 186.0 ± 2.0 μg/mL at day 21) with exosome markers detectable at day 21 (Tables 1–2), and the PCL/GEL matrix maintained high keratinocyte viability (≥90% over 14 days) (Figure S12). Collectively, these quantitative outcomes support a dual-function dressing that couples structural guidance with localized paracrine signaling to promote faster and higher-quality repair in chronic diabetic wounds.

## Supplementary Material



## Notes

(Figure S1) Representative images of DF-MSCs at passages 0, 1, 2, and 3 (Scale bar: 1000 μm); (Figure S2) representative images of osteogenic, chondrogenic, and adipogenic differentiation of DF-MSCs, stained with Alizarin Red, Alcian Blue, and Oil Red O, respectively (scale bar: 100 μm); (Figure S3) flow cytometry analysis of third-passage cells revealed high expression of MSC-positive markers (CD29:98.9%, CD105:99.2%, CD73:95.2%, CD90:97.6%) and minimal expression of MSC-negative markers (CD14:1.7%, CD34:0.6%, CD28:1.2%); (Figure S4) characterization of DF-MSCs exosomes; (Figure S5) FTIR spectra for PCL, GEL, and PCL/GEL scaffolds; (Figure S6) thermogravimetric analysis (TGA) of PCL, GEL, and PCL/GEL nanofiber scaffolds; (Figure S7) representative tensile stress–strain curves of PCL/GEL nanofiber scaffolds obtained using a TA Instruments Q800 dynamic mechanical analyzer operated in tension mode (controlled-force ramp, 0.1 N/min) at 37 °C (isothermal); (Figure S8) representative compressive stress–strain curve of PCL/GEL nanofiber scaffolds obtained using a TA Instruments Q800 dynamic mechanical analyzer operated in compression mode (controlled-force ramp, 0.1 N/min) at 37 °C (isothermal); (Figure S9) surface contact angle measurements of PCL/GEL nanofiber scaffolds; (Figure S10) water uptake rate of PCL/GEL nanofiber scaffolds; (Figure S11) in vitro biodegradation profile of a PCL/GEL nanofiber scaffold (PDF)
