# Supplementary material for: Dental Follicle-Derived Mesenchymal Stem Cell Exosome-Loaded Three-Dimensional Electrospun Poly(ε-caprolactone)/Gelatin Scaffold Accelerates Diabetic Foot Wound Healing
Source: ACS Omega. 2026 Apr 20;11(17):25171–84. doi: 10.1021/acsomega.5c11741 (PMC13150637; doi:10.1021/acsomega.5c11741)
Supplement: Supplementary file 1 [file ao5c11741_si_001.pdf]

DF-MSC Exosome–Loaded 3D Electrospun PCL/GEL Scaffold Accelerates Diabetic Foot Wound Healing

Hulya Kara Subasat[1], Deniz Genc[2,3], Osman Bulut[4], Leyla Tekin[5], Ozay Eroglu[1], Hanife Sevval Dere[1], Fatma Kuru[6], Ezgi Eren Belgin[6], Serhat Sezgin[7], Huseyin Cicek[6], Aziz Bülbül[4], Gulhan Akbaba[8], Ayse Gül[3]*

[1]Department of Energy, Molecular Nano-Materials Laboratory, Mugla Sıtkı Koçman University, 48000 Mugla, Turkiye.
[2]Faculty of Health Sciences, Mugla Sıtkı Koçman University, 48000 Mugla, Turkiye.
[3] Research Laboratories Center, Immunology and Stem Cell Laboratory, Mugla Sıtkı Koçman University, 48000 Mugla, Turkiye.
[4]Faculty of Milas Veterinary Medicine, Muğla Sıtkı Kocman University, Milas, 48000 Mugla, Turkiye.
[5]Faculty of Medicine, Department of Pathology, Mugla Sıtkı Koçman University, 48000 Mugla, Turkiye.
[6]Faculty of Science, Department of Chemistry, Mugla Sıtkı Koçman University, 48000 Mugla, Turkiye.
[7]Faculty of Dentistry, Mugla Sıtkı Koçman University, 48000 Mugla, Turkiye.
[8]Faculty of Medicine, Department of Endocrinology, Mugla Sıtkı Koçman University, 48000 Mugla, Turkiye.

[*]Corresponding author: Hulya Kara Subasat; hulyasubasat@mu.edu.tr

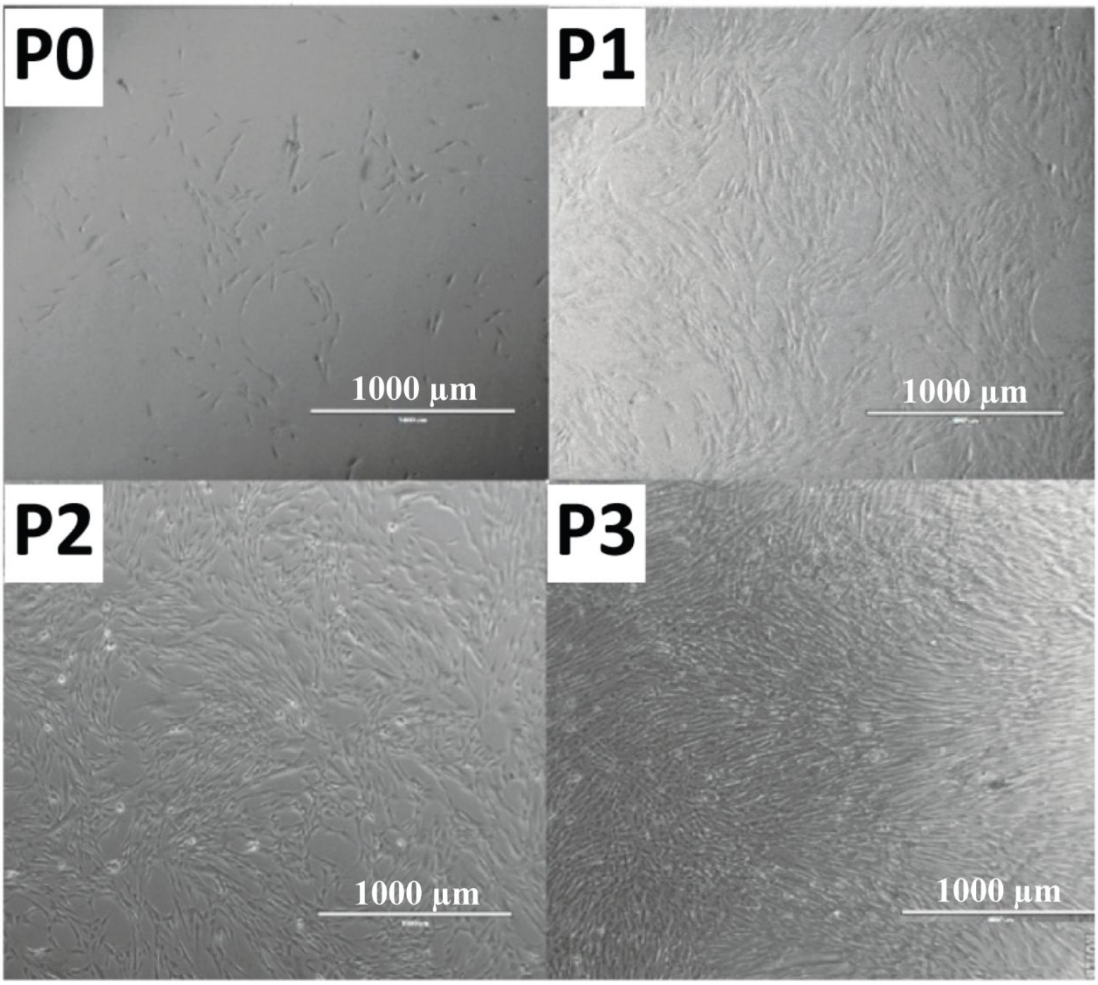

Figure S1. Representative images of DF-MSCs at passages 0, 1, 2, and 3. (Scale bar: 1000 μm).

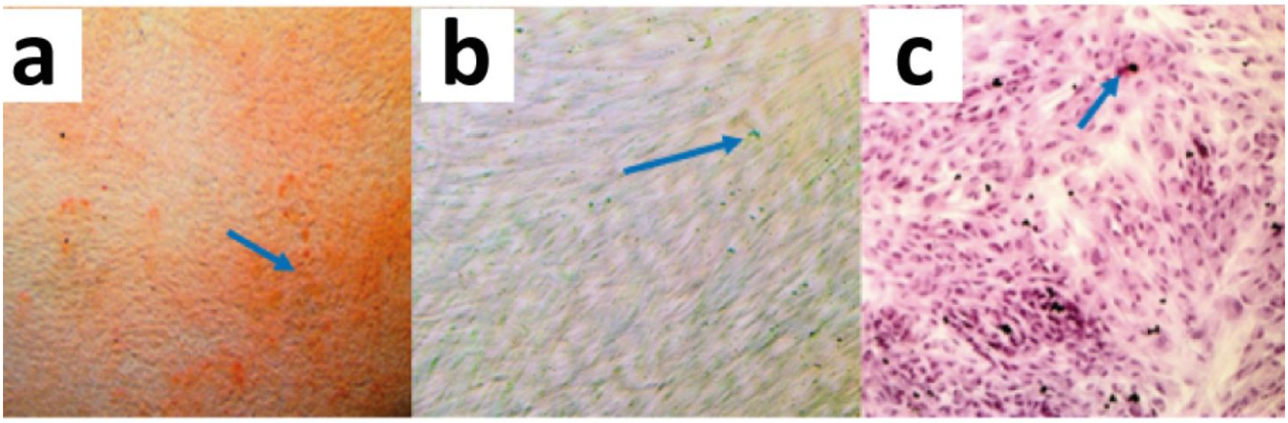

Figure S2. Representative images of osteogenic, chondrogenic, and adipogenic differentiation of DF-MSCs, stained with Alizarin Red, Alcian Blue, and Oil Red O, respectively (scale bar: 100 μm). Blue arrows represent (a) calcium deposits between the cells in osteogenic differentiated DF-MSCs, (b) proteoglycans stained with Alcian blue after chondrogenic differentiated DF-MSCs, (c) oil droplets stained with Oil Red O stain after adipogenic differentiated DF-MSCs.

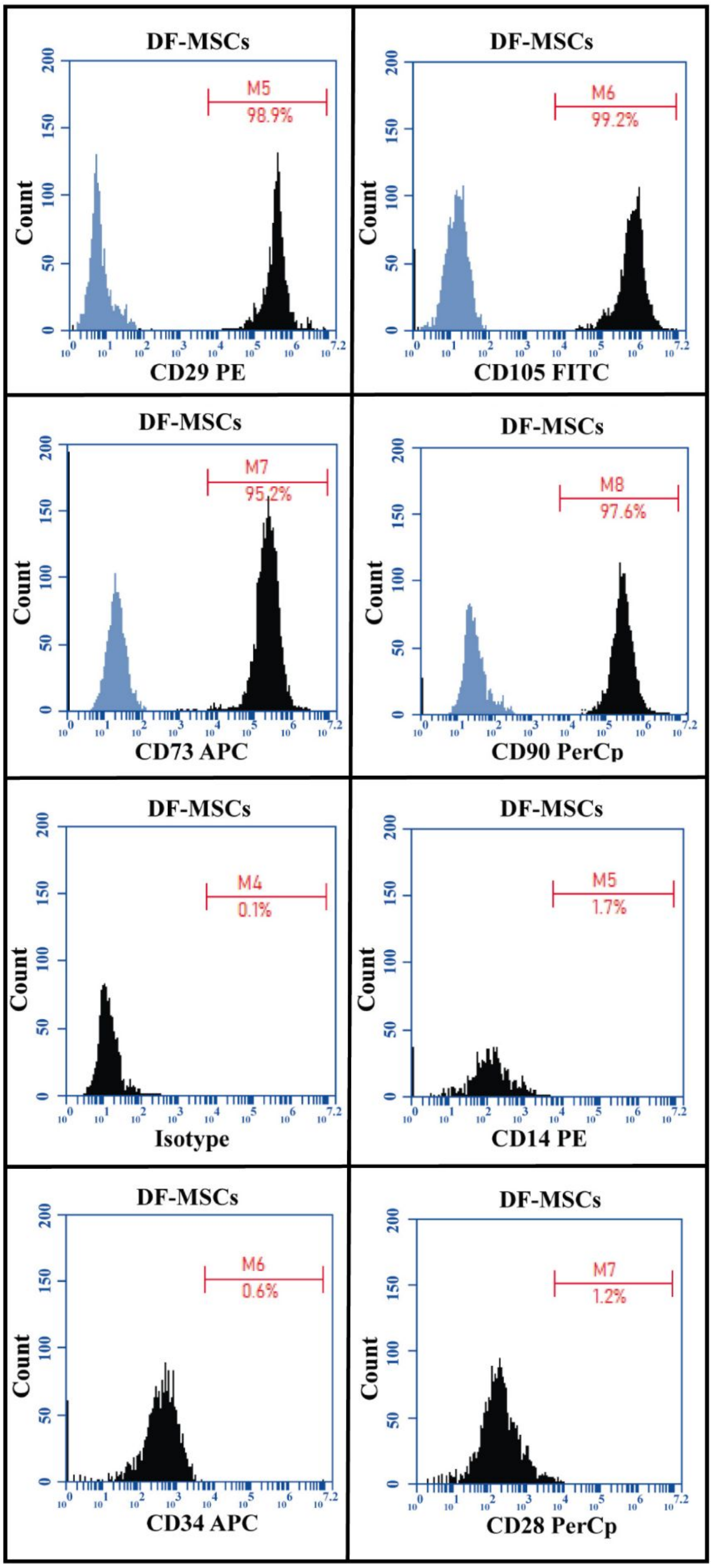

Figure S3. Flow cytometry analysis of third-passage cells revealed high expression of MSC-positive markers (CD29: 98.9%, CD105: 99.2%, CD73: 95.2%, CD90: 97.6%) and minimal expression of MSC-negative markers (CD14: 1.7%, CD34: 0.6%, CD28: 1.2%)

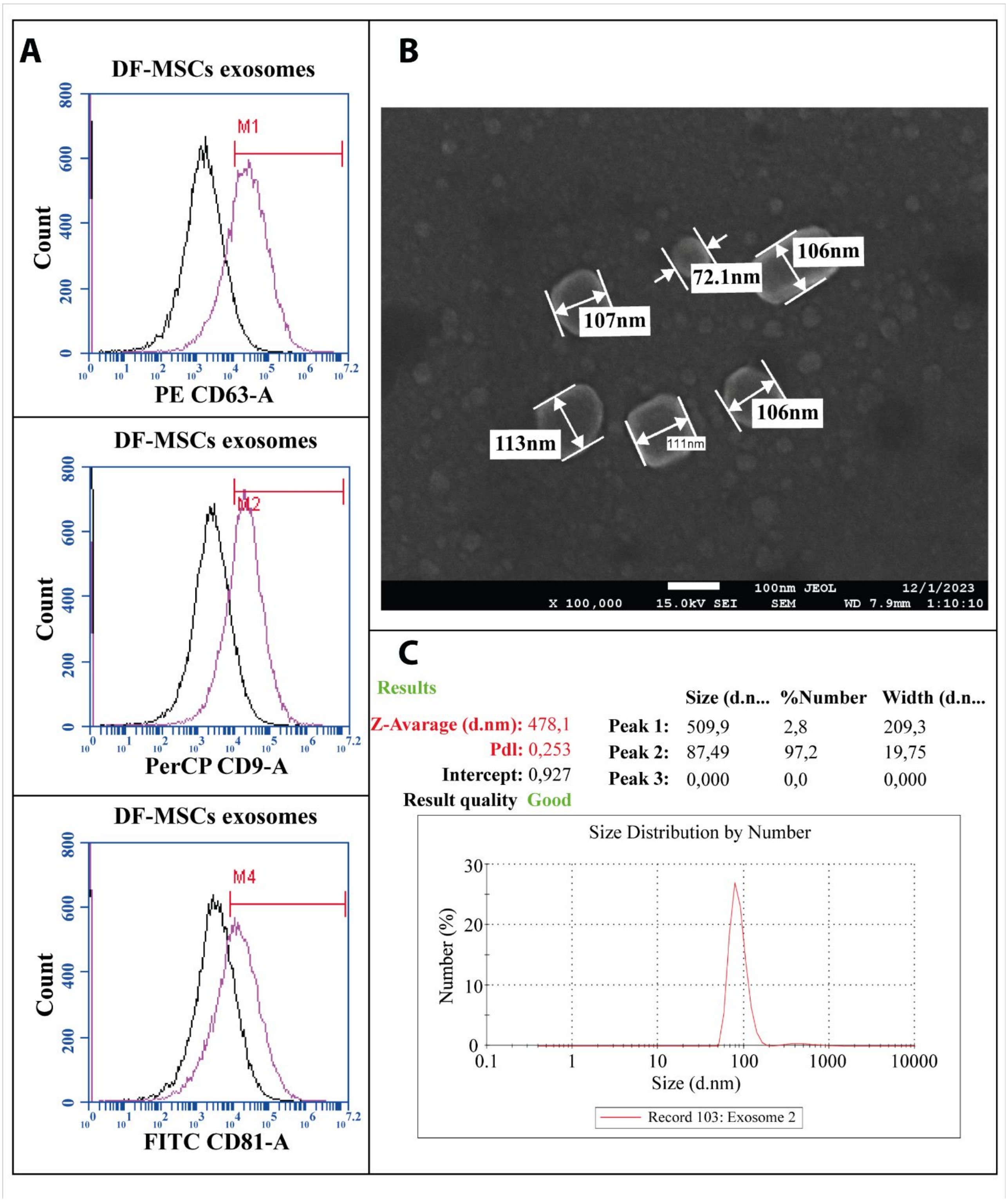

Figure S4. Characterization of DF-MSCs exosomes. (A) Representative flow cytometry analysis of positive exosome markers (CD9, CD63, CD81), with pink histograms indicating marker expression and black histograms representing isotype controls. (B) FESEM image showing particles with diameters between 30–150 nm. (C) Nanoparticle size distribution analysis indicating 97.2% of particles within the 30–150 nm range.

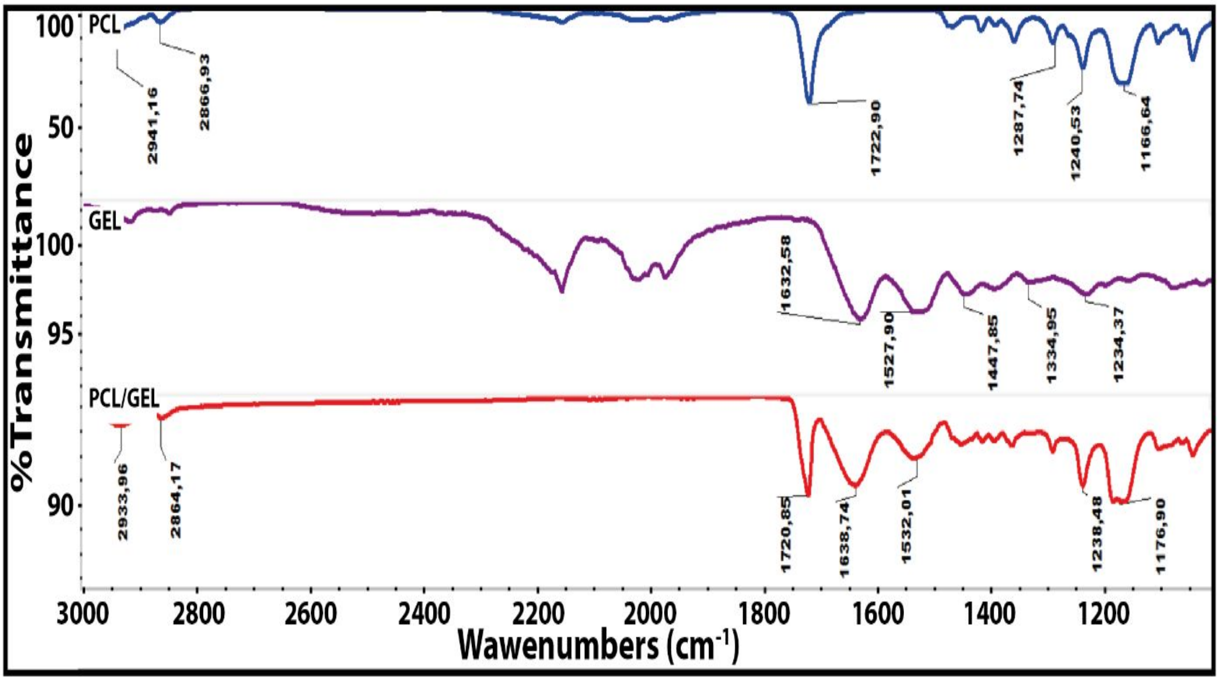

Figure S5. FTIR spectra Nanofiber scaffolds.

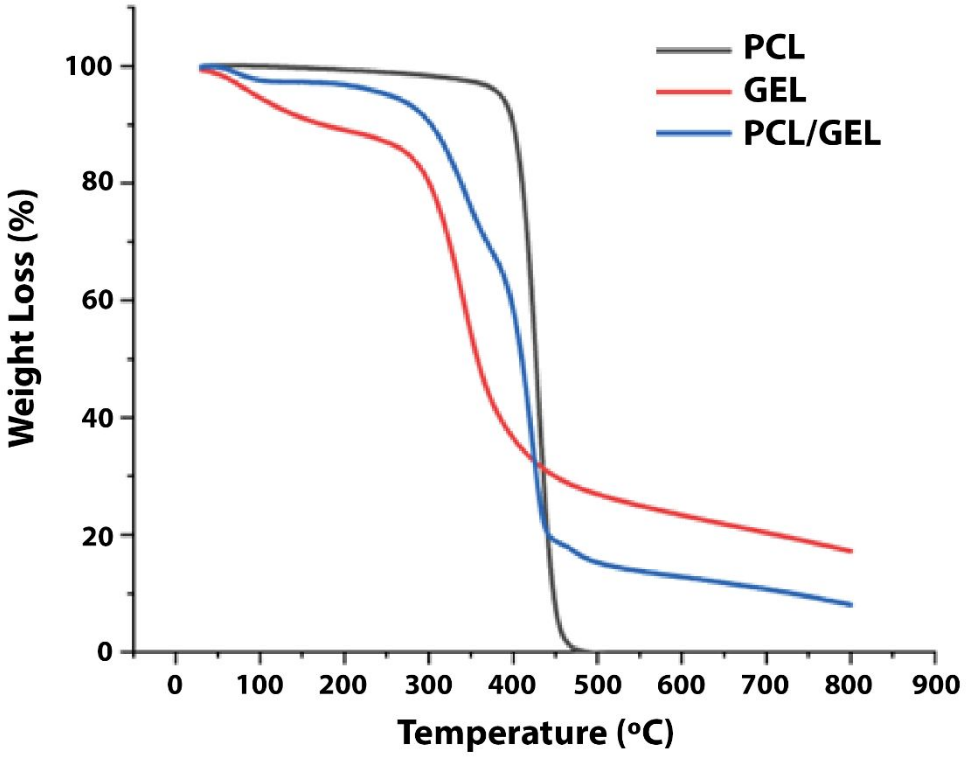

Figure S6. TGA of Nanofiber scaffolds

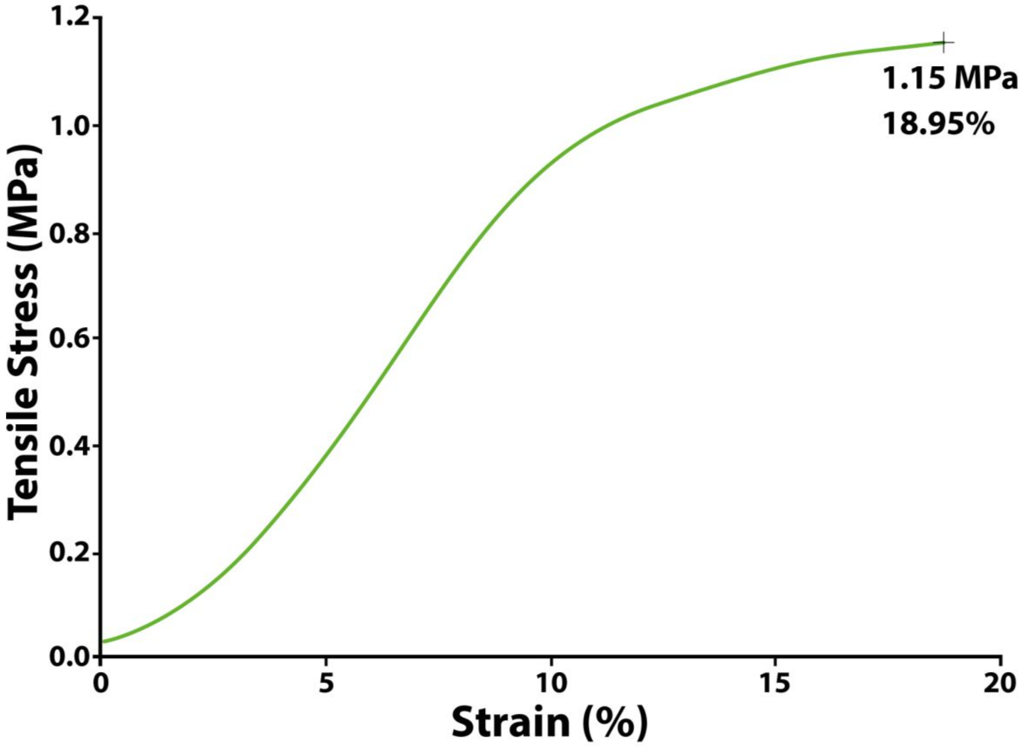

Figure S7. Representative tensile stress–strain curves of PCL/GEL nanofiber scaffolds obtained using a TA Instruments Q800 dynamic mechanical analyzer operated in tension mode (controlled-force ramp, 0.1 N/min) at 37 °C (isothermal).

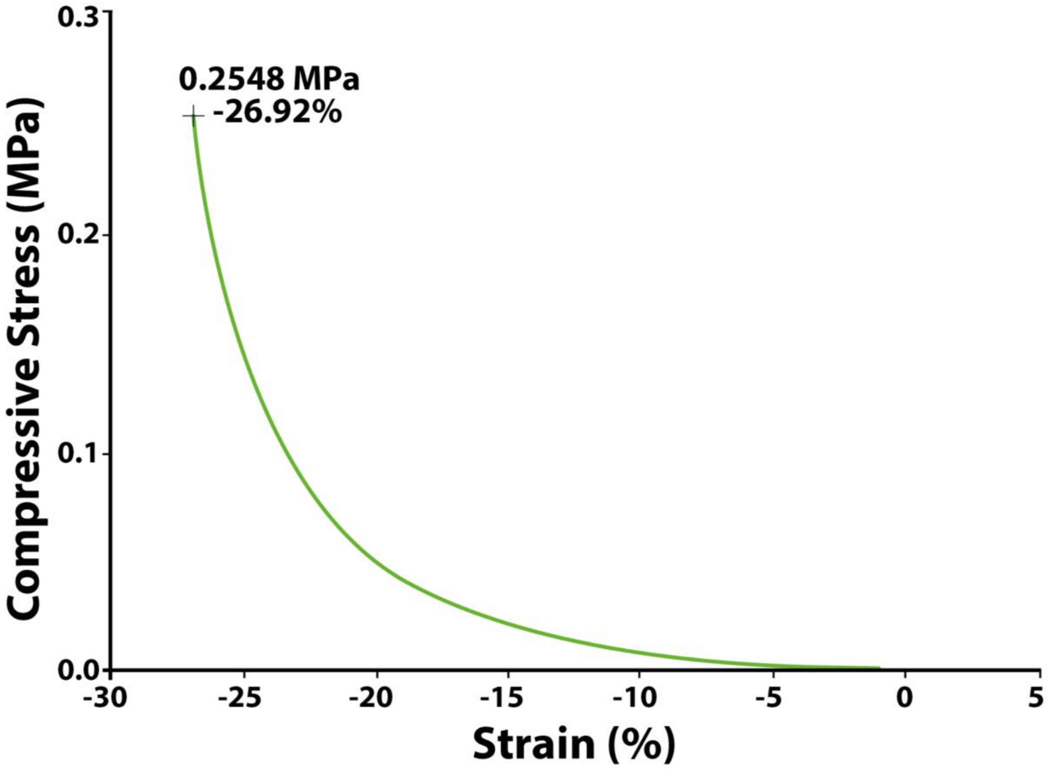

Figure S8. Representative compressive stress–strain curve of PCL/GEL nanofiber scaffolds obtained using a TA Instruments Q800 dynamic mechanical analyzer operated in compression mode (controlled-force ramp, 0.1 N/min) at 37 °C (isothermal).

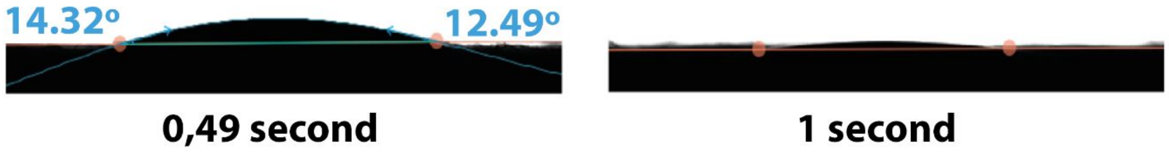

Figure S9. Surface contact angle measurements of Nanofiber scaffolds.

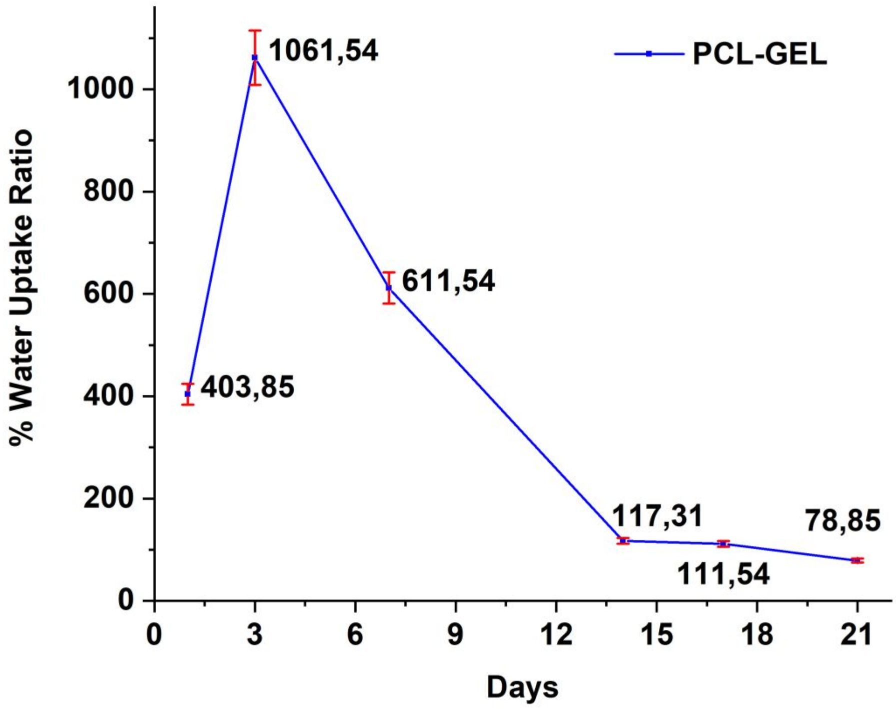

Figure S10. Water uptake rate of PCL/GEL nanofiber scaffolds. Results are presented as the average of three independent measurements (n = 3). Numerical labels on the bars indicate the percentage of water absorbed by the 3D scaffold at each time point.

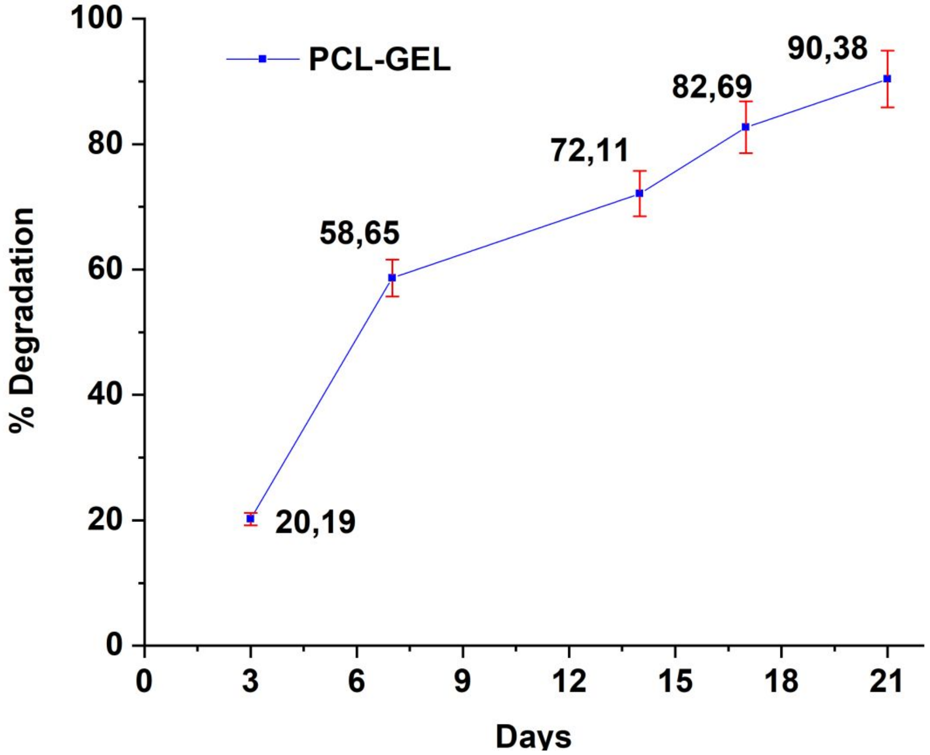

Figure S11. In vitro biodegradation profile of a PCL/GEL nanofiber scaffold. Data are presented as mean ± SD of three independent measurements (n = 3). Numerical labels indicate the percentage mass loss relative to the initial dry weight of the 3D scaffold at each time point.

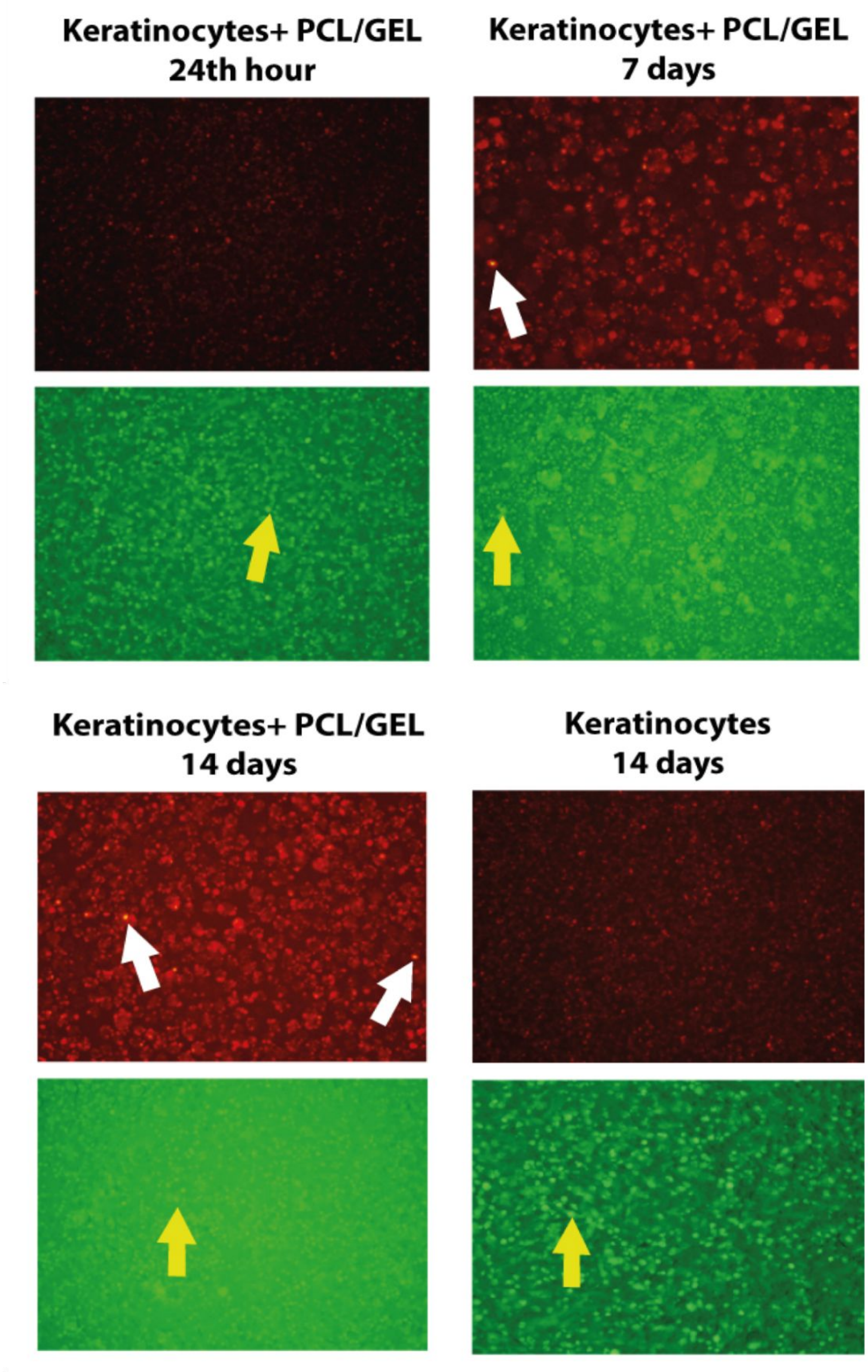

Figure S12. Fluorescent microscope images of keratinocyte cells following the biocompatibility test. Red fluorescence indicates apoptotic cells stained with Ethidium Homodimer-1 (white arrows), while green fluorescence represents viable cells stained with Calcein (yellow arrows).
